# Elevated Dietary Carbohydrate and Glycemic Intake Associate with an Altered Oral Microbial Ecosystem in Two Large U.S. Cohorts

**DOI:** 10.1158/2767-9764.CRC-22-0323

**Published:** 2022-12-05

**Authors:** Kelsey R. Monson, Brandilyn A. Peters, Mykhaylo Usyk, Caroline Y. Um, Paul E. Oberstein, Marjorie L. McCullough, Mark P. Purdue, Neal D. Freedman, Richard B. Hayes, Jiyoung Ahn

**Affiliations:** 1 Division of Epidemiology, Department of Population Health, NYU Grossman School of Medicine, New York, New York.; 2 Laura and Isaac Perlmutter Cancer Center, NYU Langone Health, New York, New York.; 3 Department of Epidemiology and Population Health, Albert Einstein College of Medicine, Bronx, New York.; 4 Department of Population Science, American Cancer Society, Atlanta, Georgia.; 5 Division of Cancer Epidemiology and Genetics, National Cancer Institute, Bethesda, Maryland.

## Abstract

The human oral microbiome is associated with chronic diseases including cancer. However, our understanding of its relationship with diet is limited. We assessed the associations between carbohydrate and glycemic index (GI) with oral microbiome composition in 834 nondiabetic subjects from the NCI Prostate, Lung, Colorectal, and Ovarian Cancer Screening Trial and the American Cancer Society Cancer Prevention Study II cohorts. The oral microbiome was characterized using 16Sv3-4 rRNA sequencing from oral mouthwash samples. Daily carbohydrate and GI were assessed from food frequency questionnaires. We used linear regression, permutational multivariate ANOVA, and negative binomial Generalized Linear Models (GLM) to test associations of diet with α- and β-diversity and taxon abundance (adjusting for age, sex, cohort, body mass index, smoking, caloric intake, and alcohol). A *q*-value (FDR-adjusted *P* value) of <0.05 was considered significant. Oral bacterial α-diversity trended higher in participants in the highest quintiles of carbohydrate intake, with marginally increased richness and Shannon diversity (*P*_trend_ = 0.06 and 0.07). Greater carbohydrate intake was associated with greater abundance of class Fusobacteriia (*q* = 0.02) and genus *Leptotrichia* (*q* = 0.01) and with lesser abundance of an Actinomyces operational taxonomic unit (*q* = 4.7E-04). Higher GI was significantly related to greater abundance of genus Gemella (*q* = 0.001). This large, nationwide study provides evidence that diets high in carbohydrates and GI may influence the oral microbiome.

**Significance:**

Taxonomic differences at the highest intake quintiles may reflect diet-induced increases in carcinogenic bacteria and decreases in protective bacteria. Genus *Leptotrichia* has been implicated in cancer and inflammatory disease, while immunostimulatory genus *Gemella* may increase inflammatory disease risk. These differences further our understanding of possible mechanisms leading to oral and systemic disease.

## Introduction

The microorganisms colonizing the human oral cavity, commonly referred to as the oral microbiome, have increasingly demonstrated a pivotal role in human health outcomes. As the ability to sequence and identify these organisms has grown, so has our understanding of their impact on human health (1–8). However, we have a limited understanding of the dietary factors influencing human oral microbiome composition.

The oral microbiome has been linked to numerous diseases, including upper oro-digestive diseases [dental caries (9), periodontitis (10), head and neck and esophageal cancer (5, 6)], and systemic diseases [rheumatoid arthritis (11), diabetes (12), cardiovascular disease (13), colorectal and pancreatic cancer (3, 14)]. While the interaction between the oral microbiome and diet is still under study, oral dysbiosis is likely influenced by dietary factors (15–17). Increased carbohydrate intake, for example, is associated with many chronic diseases (18–20), though the extent of its impact on the composition of the oral microbiome is not fully understood (20). Elevation in blood glucose levels is similarly associated with poor health and chronic disease (21–23). In addition to total carbohydrate consumption, the effect of postprandial glycemic response may also independently predict changes in microbiome composition and health outcomes. Recent studies suggest that poor glycemic control leads to an acidic oral environment due to elevated salivary glucose, thereby altering oral microbial composition (24). Glycemic index (GI) is a relative physiologic measure of the postprandial glycemic effect of a food compared with pure glucose. Diets with high GI are associated with higher levels of inflammatory biomarkers like C-reactive protein (25, 26) and an increased risk of inflammation-associated conditions like diabetes (18), arthritis (26), and cancer (27, 28). Studies in subjects with diabetes mellitus (DM), a disorder characterized by hyperglycemia, suggest that DM alters the oral microbiome through sustained inflammatory conditions (29). While connections of carbohydrates and GI with chronic disease have been extensively investigated, it is only recently, with advances in molecular sequencing technology, that the relationship of these dietary factors with the oral microbial ecosystem can be comprehensively studied, potentially revealing shared mechanisms that increase disease risk.

In an investigation of diet-oral microbial relationships, we assessed the associations of carbohydrate intake and GI with the oral microbiota in a large cross-sectional analysis of 834 nondiabetic U.S. adults from two U.S. cohort studies, the NCI Prostate, Lung, Colorectal, and Ovarian Cancer Screening Trial (PLCO) and the American Cancer Society (ACS) Cancer Prevention Study II (CPS-II).

## Materials and Methods

### Study Population

Two large cohort studies, the NCI PLCO (30) and ACS CPS-II (31), served as the source for all subjects in the current analysis. Both cohorts included adult men and women from the United States and collected baseline demographic, medical, and lifestyle data. Participants were followed prospectively to determine the occurrence of cancer and to obtain updated medical and lifestyle data. In addition to baseline questionnaires, oral wash samples were collected from a subset of participants in each cohort.

All subjects included in the current analyses were originally selected from the PLCO and CPS-II cohorts as cases or controls for collaborative nested case–control studies of the oral microbiome in relation to two cancers (head and neck cancer and pancreatic cancer; refs. 3, 4). Cases were participants who developed one of these two types of cancers at any point after collection of the oral wash samples (time from sample collection to diagnosis ranged up to 12 years). Age- and sex-matched controls were selected by incidence density sampling among cohort members who provided an oral wash sample and had no cancer prior to selection.

Because the oral microbiome assays took place at different times for the pancreas study and the head and neck study, four separate datasets were assembled for this analysis: PLCO-a (*n* = 261 PLCO participants in the head and neck study), PLCO-b (*n* = 400 PLCO participants in the pancreas study), CPS-II-a (*n* = 203 CPS-II participants in the head and neck study), and CPS-II-b (*n* = 340 CPS-II participants in the pancreas study), for a total of 1,204 subjects across all cohorts. For this study, we excluded subjects with a self-reported history of diabetes diagnosis (*n* = 115), as patients with endocrine disorders such as type 2 diabetes often consume carbohydrate-modified diets (32, 33). Previous studies show that agreement between self-reported diabetes and medical record data is high (97.2%; ref. 34). We further excluded participants based on the following criteria: missing smoking status or food frequency questionnaire (FFQ) data (*n* = 146), subjects whose microbiome assay sequencing failed (*n* = 10), implausible self-reported daily energy intake (defined as <500 or >4,000 kcal/day; *n* = 248), and low library depth (one participant with 1,516 sequence reads; *n* = 1), leaving a final population of 834 (PLCO *n* = 441, CPS-II *n* = 393; Supplementary Fig. S1) for the current cross-sectional analysis (these reasons could overlap). Written informed consent was obtained from all study participants, and all protocols were conducted in accordance with the U.S. Common Rule and approved by the New York University Grossman School of Medicine Institutional Review Board.

### Carbohydrates and GI

Carbohydrate and GI intake were assessed using validated FFQs in each cohort. Questionnaires were administered to each subject prior to the collection of baseline oral wash samples. In the PLCO, a 137-item FFQ evaluated dietary intake in the 12 months preceding study entry (35, 36). Subjects’ daily carbohydrate values (g/day) were calculated using the frequency of each consumed food item multiplied by the nutrient value of the sex-specific portion size (37). GI values were added to the nutrient database using the methods described for the NCI Diet History Questionnaire (38). Briefly, GI values were associated with individual foods from the approximately 4,200 foods defined in the Continuing Survey of Food Intakes by Individuals, condensed into 225 nutritionally similar groupings (35). In the CPS-II, a 152-item FFQ was administered to subjects in 1999 to assess dietary intake over the past year (39). Daily carbohydrate values (g/day) were obtained using estimations of daily nutrient intake from the FFQ and GI values were added to the nutrient database for individual food items using published glycemic response measures as described previously (40). In this analysis, we evaluated carbohydrates and GI as continuous and categorical variables. Continuous values were scaled to represent a 1 SD unit increase; quintiles were calculated from the full pooled dataset (*n* = 834) for categorical analysis.

### Oral Wash Sample Collection

Baseline oral wash samples were obtained by asking participants to swish with 10 mL of Scope mouthwash (P&G) for 30 seconds (41) and expectorate in a sample collection tube (30, 31). Specimens were then stored at −80°C in each cohort's biorepository prior to sequencing. This method of collection is comparable with that of fresh frozen saliva for assessment of oral microbiome composition (42).

### Microbiome Assay

Genomic DNA from oral bacteria was extracted from oral wash samples using the MoBio PowerSoil DNA Isolation Kit (Qiagen). 16Sv3-4 rRNA gene sequencing was performed on the extracted bacterial DNA and gene amplicon libraries were generated as reported previously (15, 43). Briefly, libraries were created to allow for sequencing covering variable regions V3 to V4 (Primers: 347F-5′GGAGGCAGCAGTAAGGAAT-3′ and 803R- 5′CTACCGGGGTATCTAATCC-3′). For 16Sv3-4 rRNA gene amplification preparation, 5 ng of genomic DNA was used as the template in 25 μL of PCR reaction buffer. The PCR amplicons were then purified using the Agencourt AMPure XP kit (Beckman Coulter) and purified by fluorometry using the Quant-I T PicoGreen dsDNA Assay Kit (Invitrogen; ref. 15). A total of 10^7^ molecules/μL of purified amplicons were pooled for sequencing using Roche 454 GS FLX Titanium pyrosequencing system.

### Sequence Data Processing

Following sequencing read demultiplexing, poor-quality reads were excluded using the default parameters of the microbiome bioinformatics pipeline Quantitative Insights Into Microbial Ecology (QIIME; refs. 15, 44). Reads passing quality filter parameters were clustered into operational taxonomic units (OTU) using the Human Oral Microbiome Database (HOMD) reference sequence collection (version 14.5; ref. 45) and were assigned HOMD taxonomy using QIIME script *pick_closed_reference_otus.py* (44). For this dataset of 834 subjects, there were 8,772,529 reads with a mean ± SD of 10,519 ± 2,752 and range (3,084–33,784) per sample. Good reproducibility between replicates has been demonstrated in this dataset (46).

### Statistical Analysis

Within-subject diversity (α-diversity) was calculated in 100 iterations of rarefied OTU tables of 3,000 sequence reads per sample using QIIME script *alpha_rerefaction.py* (44). This assessed α-diversity by richness, Shannon diversity index, and community evenness, with sequence depth selected by the minimum sequencing depth of our samples (min = 3,084). Linear regression was used to test the association of carbohydrates and GI with α-diversity, adjusting for age, sex, study (PLCOa, PLCOb, CPS-IIa, CPS-IIb), current smoking status, body mass index (BMI; kg/m^2^), energy intake (kcal/day), and alcohol intake (g/day). Carbohydrates and GI were modeled as both continuous and categorical variables in separate models. Because of the high correlation between total carbohydrate intake and glycemic load (GL; a measure of GI weighted by the proportion of carbohydrate in the food; ref. 47), total daily GL was not included in the statistical models to avoid multicollinearity (Pearson correlation coefficient *R* = 0.98, *P* < 2.2E-16).

Between-subject diversity (β-diversity) with respect to carbohydrate and GI intake was evaluated using permutational multivariate ANOVA (PERMANOVA) Using the “Adonis” function in the R package “vegan,” (48) PERMANOVA models included age, sex, study (PLCOa, PLCOb, CPS-IIa, CPS-IIb), current smoking status, BMI, energy intake, and alcohol intake. Carbohydrates and GI were modeled as both continuous and categorical variables in separate models. Terms are added to the PERMANOVA model sequentially, therefore separate models were run for each carbohydrate and GI quintile greater than the reference quintile (Q2–5), entering the measured quintile into the model as the second-to-last variable after the reference quintile (Q1); this ensured the quintile of interest was reflective of variation left unexplained by the other covariates in the model. In addition, we performed pairwise comparisons across carbohydrate and GI quintiles for the weighted UniFrac distance using unconstrained principal coordinate analysis (PCoA) with the R package “phyloseq” (49) and applying the Kruskal–Wallis *post hoc* test (Dunn test) to assess statistical significance when comparing quintiles within individual principle coordinates.

To test the association between carbohydrate and GI intake with microbial taxa abundance at differing taxonomic levels, negative binomial generalized linear models using DESeq2 (RRID:SCR_000154; ref. 50) were used. Raw counts of 681 OTUs were classified into 12 phyla, 26 classes, 41 orders, 71 families, 155 genera, and 549 species. To minimize the number of statistical tests conducted, rare taxa were excluded by filtering data prior to analysis to include only taxa with ≥2 sequence reads in ≥5% (*n* = 42) of subjects, resulting in 8 phyla, 17 classes, 24 orders, 42 families, 79 genera, and 293 species. DESeq2 default outlier replacement, independent filtering of low-count taxa, and filtering of count outliers were turned off. To address the large proportion of zeros in microbiome sequencing counts matrices, we calculated the taxon-level geometric mean using only positive read counts, such that taxa with zero counts are still used for downstream normalization. DESeq2’s normalization procedures have been described previously (50); briefly, the median of ratios method is used to address between-sample differences in sequencing depth and RNA composition. Of note, microbiome sequencing data are compositional, meaning the abundance of each count must be interpreted relative to the other counts within the sample (51). DESeq2’s normalization procedure is mathematically equivalent to methods designed to address compositional data (e.g., the centered log-ratio transformation; ref. 52), rendering the analysis of compositional data with DESeq2 normalization valid (51, 52). Negative binomial models in DESeq2 were adjusted for age, sex, study (PLCOa, PLCOb, CPS-IIa, CPS-IIb), current smoking status, BMI, energy intake, and alcohol intake by using a function passing the full model (including counts and all covariates) to DESeq2’s differential expression calculation argument, “DESeq()”. Carbohydrates and GI were modeled as both continuous and categorical variables in separate models. At each taxonomic level, *P* values were adjusted for FDR after removing models with maximum Cook's distance > 10 (total models removed across all comparisons: phyla *n* = 2, class *n* = 1, order *n* = 3, family *n* = 5, genus *n* = 7, species *n* = 42, OTU *n* = 50). The negative reciprocal was applied to all resulting fold changes <1.00 to reflect the negative fold-change value. Differential taxa abundance by categorical and continuous levels of carbohydrate consumption and GI was illustrated in a cladogram using GraPHIan (53).

Sensitivity analyses were conducted stratifying by BMI category (<25 kg/m^2^, ≥25 to < 30 kg/m^2^, ≥30 kg/m^2^), by study site (PLCO or CPS-II), and by sex (male and female). We also confirmed whether results were consistent when including diabetic patients, when restricting the analysis to subjects who did not subsequently develop cancer (i.e., the controls in the PLCO and CPS-II cohorts), and when assessing carbohydrates as a percent of daily calories. Finally, we assessed the impact of other dietary factors, including GL, sucrose (g/day), and fiber (g/day) on microbiome diversity and composition. A *P* value of <0.05 was considered nominally significant, and a *q*-value (FDR-adjusted *P* value) of <0.05 was considered significant after multiple comparison adjustment. All analyses were conducted using R 4.0.4.

### Data Availability

The data generated in this study are available from the Sequence Read Archive with accession number SRP133146 and SRP133149.

## Results

Carbohydrate intake was similar across both PLCO [median, 233 g/day; interquartile range (IQR): 182–282] and ACS-CPSII cohorts (median, 229 g/day; IQR: 178–284). GI was likewise similar for PLCO (median, 53.7; IQR: 51.5–55.6) and ACS-CPSII cohorts (median, 53.0; IQR: 50.9–54.8). Participants with the highest carbohydrate intake were significantly more likely to be male and have higher overall caloric intake (Table 1). Subjects with a high GI diet were similarly more likely to be male, have higher caloric intake, and lower alcohol intake overall. The Pearson correlation coefficient between carbohydrates and GI was 0.12 (*P* = 6.6E-04; Supplementary Fig. S2).

**TABLE 1:** Demographic characteristics by daily carbohydrate (a) and GI (b) **Table 1** **a**

NCI PLCO Cancer Screening Trial Cohort (*n* = 441)						ACS CPS-II Cohort (*n* = 393)					
Total carbohydrate											
Quintile 1	Quintile 2	Quintile 3	Quintile 4	Quintile 5	*P*_trend_ ^a^	Quintile 1	Quintile 2	Quintile 3	Quintile 4	Quintile 5	*P*_trend_ ^a^
*Range*											
[40.2–175]	(175–214]	(214–251]	(251–304]	(304–639]		[61.6–170]	(170–208]	(208–248]	(248–296]	(296–511]	
*N*											
89	88	88	88	88		79	78	79	78	79	
*Age (y; mean ± SD)*											
63.9 ± 5.3	63.7 ± 5.1	63.7 ± 5.2	63.7 ± 5.4	63.3 ± 5.2	0.55	73.3 ± 6.5	73.5 ± 6	73.1 ± 6.3	71.8 ± 5.7	71.9 ± 5.8	0.03
*Male (%)*											
44.9	54.5	69.3	78.4	85.2	4.8E-11	51.9	44.9	62	69.2	70.9	3.7E-04
*White (%)*											
95.5	95.5	97.7	100	93.2	0.99	100	98.7	98.7	94.9	97.5	0.08
*BMI* *^b^* *(kg/m^2^; mean ± SD)*											
26.9 ± 4.1	26.7 ± 4.1	26.8 ± 4.5	27.3 ± 4	26.6 ± 3.6	0.90	26.9 ± 5.3	25.6 ± 3.5	25.5 ± 4	25.6 ± 4.2	26.9 ± 4.5	0.84
*Current smoker (%)*											
9	8	11.4	6.8	12.5	0.55	10.1	5.1	5.1	7.7	5.1	0.39
*Alcohol (g/day; mean ± SD)*											
11.5 ± 18.9	9.2 ± 18.4	13.5 ± 34.1	11.5 ± 18.8	31.3 ± 88.6	0.52	8.5 ± 11.8	10.3 ± 14.5	13 ± 17.5	9.5 ± 13.9	10.8 ± 15.3	0.88
*Total calories (calories/day; mean ± SD)*											
926 ± 307	1026 ± 425	1011 ± 403	1128 ± 435	1386 ± 536	7.7E-11	1213 ± 246	1498 ± 286	1789 ± 279	2039 ± 287	2605 ± 472	9.9E-4
*Daily Glycemic index*											
52.6 ± 3.7	53.6 ± 3.2	53.7 ± 3.4	53.5 ± 2.5	53.7 ± 4.1	0.03	51.7 ± 3.8	52.7 ± 3	52.3 ± 3.3	53.3 ± 2.7	53.8 ± 2.6	7.5E-60
**Table 1** **b**											
**NCI PLCO Cancer Screening Trial Cohort (*n* = 441)**						**ACS CPS-II Cohort (*n* = 393)**					
**Glycemic index**											
**Quintile 1**	**Quintile 2**	**Quintile 3**	**Quintile 4**	**Quintile 5**	***P*_trend_** **^a^**	**Quintile 1**	**Quintile 2**	**Quintile 3**	**Quintile 4**	**Quintile 5**	***P*_trend_** **^a^**
*Range*											
[40.4–51]	(51–52.8]	(52.8–54.3]	(54.3–56.2]	(56.2–64.9]		[39.9–50.3]	(50.3–52.2]	(52.2–53.8]	(53.8–55.3]	(55.3–62.9]	
*N*											
89	88	88	88	88		79	78	79	78	79	
*Age (y; mean ± SD)*											
63.8 ± 5.1	63.7 ± 5.4	63.3 ± 5.5	63.8 ± 5	63.6 ± 5.3	0.85	72.6 ± 6	73.3 ± 6	72.5 ± 6	72.8 ± 6.2	72.6 ± 6.4	0.92
*Male (%)*											
57.3	63.6	69.3	68.2	73.9	0.02	44.3	67.9	65.8	56.4	64.6	0.09
*White (%)*											
97.8	98.9	95.5	97.7	92	0.05	98.7	100	97.5	94.9	98.7	0.31
*BMI* *^b^* *(kg/m^2^; mean ± SD)*											
26.9 ± 4	26.9 ± 4	26.3 ± 4	27.3 ± 4.2	26.9 ± 4	0.87	26.2 ± 5.7	25.9 ± 4.2	25.9 ± 3.5	25.8 ± 3.9	26.7 ± 4.2	0.16
*Current smoker (%)*											
10.1	5.7	9.1	12.5	10.2	0.48	6.3	7.7	5.1	7.7	6.3	0.99
*Alcohol (g/day; mean ± SD)*											
37.5 ± 87.9	10.1 ± 13.9	12.1 ± 34.7	9.6 ± 18.3	7.5 ± 14.8	8E-05	13.5 ± 17.5	13.5 ± 15.4	9 ± 14	9.7 ± 14.4	6.5 ± 10.6	0.003
*Total calories (calories/day; mean ± SD)*											
1306 ± 537	1169 ± 434	1055 ± 430	1005 ± 414	939 ± 350	3E-09	1708 ± 490	1947 ± 623	1793 ± 551	1855 ± 567	1844 ± 632	0.45
*Total carbohydrates (carbs/day; mean ± SD)*											
234 ± 98	234 ± 77	253 ± 98	251 ± 92	250 ± 88	0.04	202 ± 62	248 ± 78	238 ± 73	243 ± 80	256 ± 85	3E-04

^a^ *P* values are from Spearman correlations for continuous variables, *Χ*^2^ test for trend for categorical variables.

^b^Value for participants missing BMI (*n* = 24) imputed with cohort-specific medians.

**FIGURE 1:**
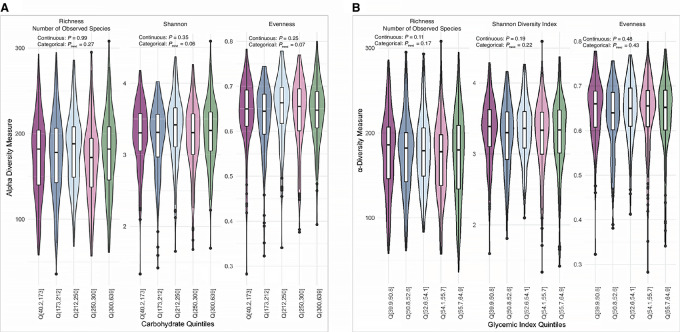
Richness, diversity, and evenness of oral microbiome by carbohydrate and GI quintiles (*n* = 834). Violin plots of α-diversity metrics across quintiles of daily carbohydrate (**A**) and GI (**B**). Plotted are median, IQRs, and the probability density of the indices at different values. *P* values are from linear regression models with specified α-diversity metric (richness, Shannon diversity index, community evenness, averaged over 100 iterations of rarefied OTU table at 3,000 sequence reads/sample) as the outcome. All models were adjusted for age, sex, study (PLCOa, PLCOb, CPS-IIa, CPS-IIb), current smoking, BMI (kg/m^2^), energy intake (kcal/day), and alcohol intake (g/day).

**FIGURE 2:**
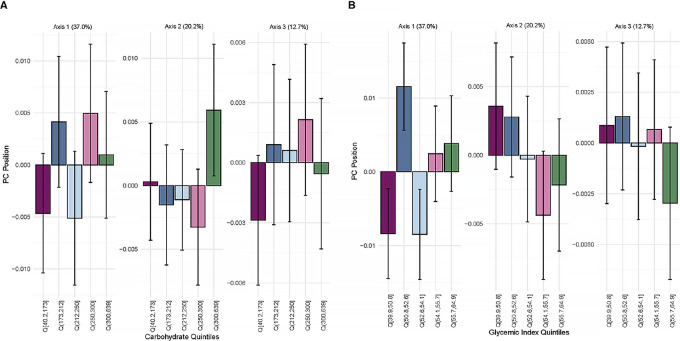
PCoA of phylogenetic distance matrices, carbohydrate and GI quintiles (*n* = 834). Bar plots of β-diversity metrics showing the means of the first, second, and third coordinates (with % variance explained by each coordinate) of PCoA for each quintile of daily carbohydrate (**A**) and GI (**B**) using weighted UniFrac phylogenetic distance matrices.

We observed a trend of higher overall α-diversity indices in participants with higher carbohydrate intake, although the results were not statistically significant (Shannon index *P*_trend_ = 0.06, Evenness *P*_trend_ = 0.07 across carbohydrate quintiles; Fig. 1A; Supplementary Table S1); greater GI was marginally associated with decreased α-diversity (richness *P* = 0.11 for continuous GI; Fig. 1B; Supplementary Table S1). In PERMANOVA analysis of the weighted UniFrac distance, β-diversity was not associated with carbohydrate intake (*P*_trend_ across quintiles = 0.71, continuous *P* = 0.38; Fig. 2A; Supplementary Table S2) or with GI (*P*_trend_ across quintiles = 0.33, continuous *P* = 0.26; Fig. 2B; Supplementary Table S2).

**FIGURE 3:**
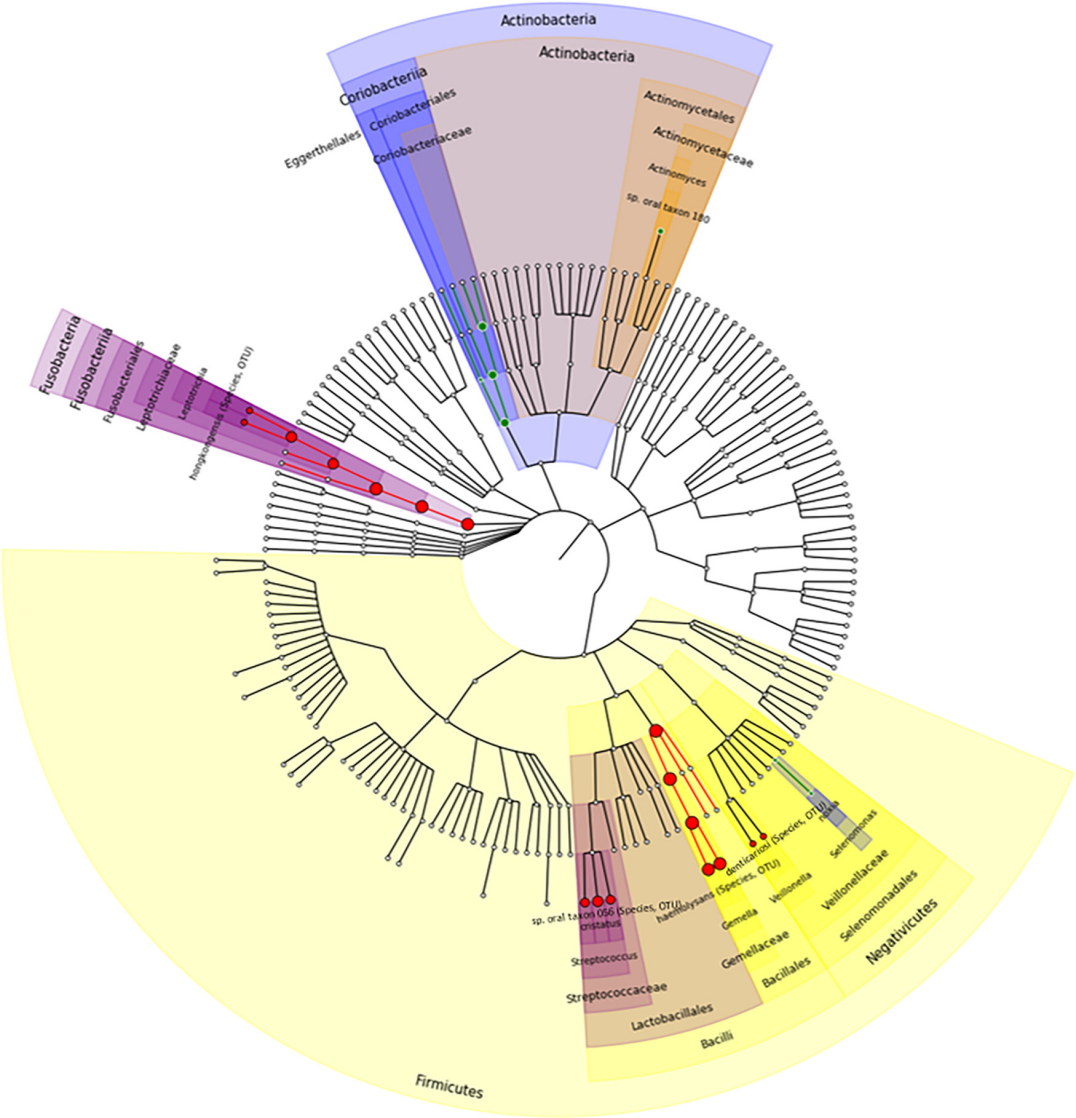
Cladogram of phylum- to species-level taxa associated with carbohydrate and GI quintiles (*n* = 834). Only taxa that were identified in the DESeq2 models as significantly different (*q* < 0.05) across quintiles or continuous carbohydrate intake or GI are labeled and colored by phylum. Red nodes indicate significant increases in taxa abundance with increased carbohydrate intake or GI; green nodes indicate significant decreases in abundance with increased carbohydrate or GI. Size of the significant nodes is proportional to the mean normalized count of the observed taxa. Taxa shaded in purple are those significantly increased with high carbohydrate; taxa shaded in orange are those significantly decreased with high carbohydrate. Taxa shaded in yellow are those significantly increased in subjects with high GI; taxa shaded in blue are those significantly decreased with high GI.

We further investigated specific taxon abundance related to carbohydrate intake and GI using negative binomial generalized linear models. Higher carbohydrate intake was associated with an increase in taxa belonging to class Fusobacteriia and its genus *Leptotrichia* (nominal *P*_trend_ = 1.5E-04, *q*-trend = 0.01 across carbohydrate quintiles). The highest quintile of carbohydrate intake had a 1.98-fold increase in species *Leptotrichia hongkongensis* [95% confidence interval (CI), 1.28–3.07] and a 2.02-fold increase in OTU *hongkongensis* (95% CI, 1.30–3.13) compared with the lowest quintile. We also observed an increase in several species of genus *Streptococcus* with continuous carbohydrate intake including *Streptococcus* oral taxon 056 (species *q* = 0.02 and OTU *q* = 0.01) and *streptococcus cristatus* OTU (*q* = 0.03). Greater carbohydrate intake was also associated with a decrease in family *Coriobacteriaceae* (*q* = 0.01) and an OTU in genus *Actinomyces* (phylum Actinobacteria), with a 2.56-fold reduction for the highest carbohydrate quintile compared with the lowest quintile for *Actinomyces* oral taxon 180 (95% CI = −4.17 to −1.59; Fig. 3; Table 2), although their abundances are low (mean normalized count = 22.3). In addition, increased GI was associated with increases in family *Gemellaceae* (nominal *P*_trend_ = 3.6E-05, *q* = 0.001 across GI quintiles; Fig. 3; Table 2) and its genus *Gemella*, including *Gemella haemolysans* species (nominal *P*_trend_ = 1.8E-04, *q*-trend = 0.02 across GI quintiles) and OTU (*P*_trend_ = 1.9E-04, *q*-trend = 0.03). The highest quintile of GI had a 1.62-fold increase of species *Gemella haemolysans* compared with the lowest quintile of intake (95% CI = 1.24–2.13).

**TABLE 2a:** Association of daily carbohydrate (a) and GI (b) as categorical (quintiles) or continuous variables with abundance of oral microbial taxa, PLCO and CPS-II cohorts (*n* = 834)^a^ **Table 2** **a**

Categorical								Continuous^b^		
Fold change^c^,^d^ (95% CI)								Fold change^c^^,^^d^ (95% CI)		
Total carbohydrates										
*Mean normalized count*	Q1	Q2	Q3	Q4	Q5	*P*_trend_ ^e^	*q*-trend^e^	Grams per day	*P*	q
Fusobacteria; Fusobacteriia; Fusobacteriales; *Leptotrichiaceae; Leptotrichia* (Genus)										
121	*Ref*	1.11 (−1.10 to 1.37)	1.28 (1.04–1.58)	1.28 (1.03–1.60)	1.65 (1.28–2.13)	1.5E-04	0.01	1.18 (1.08–1.29)	1.8E-04	0.01
Fusobacteria; Fusobacteriia; Fusobacteriales; *Leptotrichiaceae; Leptotrichia; hongkongensis* (Species)										
12.3	*Ref*	1.08 (−1.32 to 1.53)	1.08 (−1.33 to 1.55)	1.36 (−1.08 to 2.00)	1.98 (1.28– 3.07)	0.004	0.18	1.34 (1.15–1.56)	1.3E-04	0.02
Firmicutes; Bacilli; Lactobacillales; *Streptococcaceae; Streptococcus; sp._oral_taxon_056* (Species)										
59.1	*Ref*	1.10 (−1.14 to 1.37)	1.03 (−1.22 to 1.30)	1.32 (1.04–1.68)	1.5 (1.14–1.98)	0.003	0.18	1.20 (1.09–1.32)	1.6E-04	0.02
Firmicutes; Bacilli; Lactobacillales; *Streptococcaceae; Streptococcus; cristatus* (OTU)										
198	*Ref*	1.05 (−1.12 to 1.23)	1.04 (−1.14 to 1.23)	1.13 (−1.05 to 1.35)	1.29 (1.05–1.57)	0.02	0.32	1.13 (1.06–1.21)	4.3E-04	0.03
Actinobacteria; Actinobacteria; Actinomycetales; *Actinomycetaceae*; *Actinomyces*; *sp. oral taxon 180* (OTU)										
22.4	*Ref*	−1.05 (−1.56, 1.39)	−1.20 (−1.82, 1.23)	−1.92 (−2.94, −1.27)	−2.56 (−4.17, −1.59)	1.1E-05	0.003	−1.49 (−1.75, -1.27)	1.6E-06	4.7E-04
**Table 2** **b**										
**Categorical**								**Continuous** **^b^**		
**Fold change** **^c^** **^,^** **^d^** **(95% CI)**								**Fold change** **^c^** **^,^** **^d^** **(95% CI)**		
**Glycemic index**										
***Mean normalized count***	**Q1**	**Q2**	**Q3**	**Q4**	**Q5**	***P*_trend_** **^e^**	***q*-trend** **^e^**	**Grams per day**	***P***	***q*-value**
Firmicutes; Bacilli; Bacillales; *Gemellaceae; Gemella* (Genus)										
337	*Ref*	1.21 (−1.02 to 1.49)	1.07 (−1.15 to 1.32)	1.51 (1.22–1.86)	1.44 (1.16–1.79)	8.5E-05	0.01	1.12 (1.04–1.20)	0.002	0.15
Firmicutes; Bacilli; Bacillales; *Gemellaceae; Gemella*; *haemolysans* (Species)										
235	*Ref*	1.42 (1.09–1.85)	1.28 (−1.02 to 1.67)	1.73 (1.32–2.25)	1.62 (1.24–2.13)	1.8E-04	0.02	1.13 (1.04–1.24)	0.01	0.33
Firmicutes; Negativicutes; Selenomonadales; *Veillonellaceae; Veillonella; denticariosi* (Species)										
3.48	*Ref*	1.82 (1.2–2.77)	1.92 (1.27–2.93)	2.72 (1.79–4.13)	2 (1.31–3.07)	1.8E-04	0.02	1.3 (1.13–1.50)	1.9E-04	0.05
Firmicutes; Negativicutes; Selenomonadales; *Veillonellaceae*; *Selenomonas*; *noxia* (OTU)										
0.85	*Ref*	−1.56 (−2.70 to 1.1)	−1.61 (−2.78 to 1.06)	−2.38 (−4.17 to -1.39)	−2.94 (−5.26 to 1.69)	4.1E-05	0.01	−1.45 (−1.73,-1.20)	7.8E-05	0.02

^a^Taxa included in the table were associated with daily carbohydrate or GI at *q*-trend <0.05 for categorical variables or *q* value < 0.05 for continuous variables.

^b^Continuous values scaled by 1 SD unit increase.

^c^DESeq2 models adjusted for age, sex, study (PLCOa, PLCOb, CPS-IIa, CPS-IIb), current smoking, BMI (kg/m^2^), energy intake (kcal/day), and alcohol intake (g/day).

^d^Fold change <1.00 represented as the negative reciprocal.

^e^Trend tests across quintiles were calculated by entering the categorical variables into the models as continuous terms.

In stratified analyses, we examined the effect of carbohydrates and GI on the oral microbiome according to categories of BMI status (normal <25, *n* = 328; overweight 25–30, *n* = 358; and obese BMI >30, *n* = 148; Supplementary Tables S3A–S5A). The relationships were largely consistent across BMI categories, with significantly lower α-diversity with elevated carbohydrate and GI (Supplementary Table S3A) and no association with β-diversity (Supplementary Table S4A). Taxa in subjects with elevated BMI (≥25) were moderately distinct from lower BMI categories, with differences in the obese subjects potentially due to the smaller sample size (*n* = 148; Supplementary Table S5A).

Findings in carbohydrates and GI are consistent in both PLCO (*n* = 441) and ACS-CPSII (*n* = 393) cohorts (Supplementary Tables S3B–S5B), and in men (*n* = 528; Supplementary Tables S3C–S5C). The stratified analysis focusing on women (*n* = 306) showed consistency with the main results, except for significant decreases in α-diversity at the highest carbohydrate quintile (Shannon index *P*_trend_ = 0.04; Evenness *P*_trend_ = 0.02). The findings in the full cohort including diabetics (*n* = 938) were relatively unchanged, with modest differences in α- and β-diversity in the carbohydrate analysis (Supplementary Tables S3D–S5D). α- and β-diversity metrics were similar when restricting to PLCO and CPS-II controls (*n* = 543), but these subjects had relatively distinct taxa, particularly in the GI analysis, in which no taxa passed the initial *q* < 0.05 threshold for significant differences across intake levels. No significant differences from the main analysis were observed when assessing carbohydrates as a percent of daily calories (Supplementary Tables S3F–S5F).

Finally, we assessed the association between the microbiome and other similar dietary exposures: GL (the composite measure of GI and carbohydrates) and specific types of carbohydrates (sucrose and fiber; Supplementary Tables S3G–S5G). α- and β-diversity were similar across all three exposures and compared to our main analysis. When comparing the differential abundance results with our main analysis, there were no shared taxa at the family level when assessing sucrose intake, while the results in the fiber analysis showed some similarities in taxa to both the carbohydrate and GI analysis. Our assessment of GL and oral microbial diversity identified a lower abundance of genus *Actinomyces* (FDR-adjusted *q* < 0.0005) with increased GL, consistent with the association we observed with carbohydrate intake, and distinct from the findings based on GI intake.

## Discussion

To our knowledge, this is the first examination of the association of carbohydrate intake and GI with the oral microbiome. In a large, cross-sectional study of American adults, this analysis demonstrated differentials in the diversity of the oral microbiome characterized by increased Fusobacteriia and *Leptotrichiaceae* and decreased abundance of *Actinomyces* with greater carbohydrates, and higher abundance of *Gemellaceae* and *Gemella* with elevated GI.

Our findings may be a result of diet-induced disruptions to the composition of the oral microbiome. Carbohydrate intake has been hypothesized to affect the oral microbiome, with a shift in disease-promoting microbiota associated with the transition to a diet rich in carbohydrates in late hunter-gatherer and early agrarian societies (54). A “constant supply” (55) of carbohydrates is necessary for dysbiosis-mediated oral diseases like dental caries and periodontal disease (55). The proposed mechanism for this relationship is the microbial production of glucans as a result of carbohydrate intake, which disrupts the glyco-mediated salivary immune response and prevents the removal of harmful bacteria (55, 56). In addition to increasing the risk of oral disease, this pathogenic dysbiosis then promotes an inflammatory immune response (55, 57). This inflammation stimulates cytokine-induced epithelial cell proliferation (58), potentially leading to unchecked cell growth and, ultimately, cancer (59). Indeed, the risk of developing cancer is increased two to five times in those with periodontitis (7). Dysbiosis of the oral microbiome has also been linked to several chronic inflammatory conditions such as diabetes, systemic lupus erythematosus, and rheumatoid arthritis (60). Similarly, higher carbohydrate intake is also a risk factor for autoimmune conditions such as rheumatoid arthritis (26) and is a key driver of inflammation (25).

The observed increase in genus *Leptotrichia* in those with increased carbohydrate intake is noteworthy given the established connection between the prevalence of order Fusobacteriales and elevated risk of periodontal disease (61), oral (62), pancreatic (63), and colorectal cancers (64, 65), and inflammatory conditions such as ulcerative colitis (66). While order Fusobacteriales, including genus *Leptotrichia*, are typically considered to be oral anaerobic bacteria, there are strong associations with these bacteria at distal disease sites, suggesting the potential for bacterial translocation from oral to intestinal sites (67, 68). Colorectal cancer in particular is strongly associated with genus *Fusobacterium*, and studies show significant co-occurrence of genus *Leptotrichia* in *Fusobacterium*-positive tumors (67, 69). *Fusobacteriae* invade host epithelial and oral mucosal cells through their surface adhesion molecule, *Fusobacterium* adhesin A (FadA), activating β-catenin signaling pathways responsible for inflammation and oncogenic cellular proliferation. Species within genus *Leptotrichia* also possess FadA (70), and the genetic and functional similarities between these two genera may be due to horizontal gene transfer between families *Fusobacteriaceae* and *Leptotrichiaceae* (66). Consumption of proinflammatory diets (which include carbohydrate-rich foods such as refined grains, beer, and pizza) is associated with *Fusobacteria*-positive colorectal cancer, but not with tumors lacking these bacteria (71). This suggests that diet-induced alterations to the microbiome may increase the prevalence of disease-promoting bacteria. While this hypothesis is mechanistically plausible given the activation of oncogenic pathways by FadA, it may be that the cancer alters the host microbiota instead (72). Other mechanisms, including chance, are also possible. Further epidemiologic study is warranted to confirm the directionality of this relationship, the potential bacterial translocation of these oral microbes, and the function of *Leptotrichia*-derived FadA in light of our observation of elevated levels of oral *Leptotrichia* with increased carbohydrate intake.

We found an association between elevated carbohydrates and decreased abundance of *Actinomyces oral taxon 180*. Previous research in the Southern Community Cohort Study indicated that decreased levels of oral genus *Actinomyces* (phylum Actinobacteria) are associated with an increased risk of diabetes (73). A possible role of oral Actinobacteria in diabetes may be through the inhibition by this bacteria of *N*-acetyl-beta-D-glucosaminidase (73, 74), an enzyme linked to decreased glucose metabolism and diabetes risk (74). A decrease in *Actinomyces* related to diet-induced dysbiosis may reduce glycemic control, suggesting avenues for further study of a potential mechanism linking carbohydrate intake, oral microbial composition, and risk of systemic disease.

We also found that higher GI was related to greater abundance of genus *Gemella* (phylum Firmicutes). Other research has shown a higher abundance of *Gemella* in subjects with inflammatory conditions such as obesity (73), diabetes (12, 73), and inflammatory bowel disease (75). The majority of bacteria under the Firmicutes phylum are Gram positive, including *Gemella*, and the peptidoglycan component of Gram-positive bacterial cell walls is a potent stimulator of proinflammatory cytokines (76). A hypothesis for the potential pathogenic relationship between increases in *Gemella* and inflammatory diseases is through the promotion of low-grade inflammation from immune stimulation by Gram-positive peptidoglycans (77), although these studies have primarily assessed the presence of *Gemella* in the gut microbiome. The overabundance of oral *Gemella* associated with elevated GI in our sample suggests the presence of diet-induced dysbiosis, although the link between *Gemella* abundance and oral inflammation has not been fully explored.

Our sensitivity analyses were largely consistent with our main findings, with several key differences. Stratification by sex identified significantly decreased α-diversity in women with the highest carbohydrate intake, recapitulating findings from a recent study of the association between subgingival microbiota and carbohydrate intake in a large cohort of postmenopausal women (78). As in our study of intraindividual salivary microbiome differences, the authors identified an inverse relationship between carbohydrate consumption and subgingival α-diversity, highlighting concordance between these two similar, yet distinct, sources of oral microbiome data.

The consistent results in our cohort for a lack of difference in α- and β-diversity between carbohydrate, sucrose, and fiber intake, as well as GI and GL, suggest that the type of carbohydrate consumed may not meaningfully influence oral microbial diversity across intake quintiles. A sensitivity analysis assessing the association of GL and the oral microbiome showed consistency with the findings of taxonomic diversity in our carbohydrate analysis, and no overlap with our GI results. Given the high correlation between GL and carbohydrates in our dataset, these results highlight the utility of analyzing the components of the composite GL variable (namely, carbohydrates and GI) individually.

Intriguingly, none of our key taxonomic findings in *Leptotrichia*, *Gemella*, or *Actinomyces oral taxon 180*—all of which point to potentially pathogenic mechanisms due to oral dysbiosis—were identified when restricting the analysis to the subset of patients in the PLCO and CPS-II cohorts who did not subsequently develop cancers (i.e., the controls in the nested case–control analysis). This points to avenues for further prospective research on the effects of diet-induced changes to the oral ecosystem which may be related to subsequent disease development. However, there is also the potential for reverse causation, wherein undetected precancerous or early-stage cancers contribute to the observed taxonomic differences. Longitudinal studies with serial collections of oral wash samples can be used to interrogate these potential causal mechanisms and determine the direction of causality.

Among the strengths of this study were its large sample size (*n* = 834) and the ability to obtain comprehensive phylogenetic information for the composition of the oral microbiome using 16Sv3-4 rRNA sequencing, mapped to the HOMD reference sequence collection (45). In addition, the detailed demographic and FFQs allowed us to control for known confounders of the composition of the oral microbiome, such as smoking and alcohol consumption. Study limitations include its cross-sectional design, which limits our ability to make causal inferences or directly assess the association with subsequent disease development, although it is more plausible that diet influences the oral microbiome rather than the reverse having occurred. Our findings are also susceptible to inherent measurement error associated with diet assessment from self-administered FFQ. We did not have information on the oral health of participants, nor their oral hygiene practices, which may be another important confounder of oral microbiome composition. We also sampled the average bacterial composition of the mouth from oral wash samples rather than specific sites in the mouth, which vary in microbial composition. Finally, the study population was primarily white, which limits the generalizability of these results to other, more diverse populations.

In conclusion, in this large study, we showed for the first time that high carbohydrate intake is related to increased abundance of *Leptotrichia* and decreased abundance of *Actinomyces oral taxon 180* and that elevated GI is related to increased abundance of *Gemella.* Further studies are warranted to replicate these findings and assess potential underlying molecular pathways and links to disease.

## Supplementary Material

Figure S1 Population flow diagram

Figure S2 Carbohydrate and GI Pearson correlation

Table S1 Carbohydrate and GI Alpha diversity

Table S2 Carbohydrate and GI Beta diversity

Table S3 Sensitivity analyses, alpha diversity

Table S4 Sensitivity analyses, beta diversity

Table S5 Sensitivity analyses, taxon abundance

## References

[1] Irfan M , Delgado RZR, Frias-Lopez J. The oral microbiome and cancer. Front Immunol 2020;11:591088. 33193429 10.3389/fimmu.2020.591088 PMC7645040

[2] Sun J , Tang Q, Yu S, Xie M, Xie Y, Chen G, . Role of the oral microbiota in cancer evolution and progression. Cancer Med 2020;9:6306–21. 32638533 10.1002/cam4.3206 PMC7476822

[3] Fan X , Alekseyenko AV, Wu J, Peters BA, Jacobs EJ, Gapstur SM, . Human oral microbiome and prospective risk for pancreatic cancer: a population-based nested case-control study. Gut 2018;67:120–7. 27742762 10.1136/gutjnl-2016-312580 PMC5607064

[4] Hayes RB , Ahn J, Fan X, Peters BA, Ma Y, Yang L, . Association of oral microbiome with risk for incident head and neck squamous cell cancer. JAMA Oncol 2018;4:358–65. 29327043 10.1001/jamaoncol.2017.4777 PMC5885828

[5] Wang L , Ganly I. The oral microbiome and oral cancer. Clin Lab Med 2014;34:711–9. 25439271 10.1016/j.cll.2014.08.004

[6] Peters BA , Wu J, Pei Z, Yang L, Purdue MP, Freedman ND, . Oral microbiome composition reflects prospective risk for esophageal cancers. Cancer Res 2017;77:6777–87. 29196415 10.1158/0008-5472.CAN-17-1296 PMC5726431

[7] Tuominen H , Rautava J. Oral microbiota and cancer development. Pathobiology 2021;88:116–26. 33176328 10.1159/000510979

[8] Willis JR , Gabaldón T. The human oral microbiome in health and disease: from sequences to ecosystems. Microorganisms 2020;8:308. 32102216 10.3390/microorganisms8020308 PMC7074908

[9] Koo H , Bowen WH. Candida albicans and Streptococcus mutans: a potential synergistic alliance to cause virulent tooth decay in children. Future Microbiol 2014;9:1295–7. 25517895 10.2217/fmb.14.92

[10] Jorth P , Turner KH, Gumus P, Nizam N, Buduneli N, Whiteley M. Metatranscriptomics of the human oral microbiome during health and disease. mBio 2014;5:e01012–14. 24692635 10.1128/mBio.01012-14 PMC3977359

[11] Roszyk E , Puszczewicz M. Role of human microbiome and selected bacterial infections in the pathogenesis of rheumatoid arthritis. Reumatologia 2017;55:242–50. 29332963 10.5114/reum.2017.71641 PMC5746635

[12] Casarin RC , Barbagallo A, Meulman T, Santos VR, Sallum EA, Nociti FH, . Subgingival biodiversity in subjects with uncontrolled type-2 diabetes and chronic periodontitis. J Periodontal Res 2013;48:30–6. 22762355 10.1111/j.1600-0765.2012.01498.x

[13] Chhibber-Goel J , Singhal V, Bhowmik D, Vivek R, Parakh N, Bhargava B, . Linkages between oral commensal bacteria and atherosclerotic plaques in coronary artery disease patients. NPJ Biofilms Microbiomes 2016;2:7. 28649401 10.1038/s41522-016-0009-7 PMC5460270

[14] Kato I , Vasquez AA, Moyerbrailean G, Land S, Sun J, Lin HS, . Oral microbiome and history of smoking and colorectal cancer. J Epidemiol Res 2016;2:92–101. 28111632 10.5430/jer.v2n2p92 PMC5241083

[15] Peters BA , McCullough ML, Purdue MP, Freedman ND, Um CY, Gapstur SM, . Association of coffee and tea intake with the oral microbiome: results from a large cross-sectional study. Cancer Epidemiol Biomarkers Prev 2018;27:814–21. 29703763 10.1158/1055-9965.EPI-18-0184 PMC6889868

[16] Kato I , Vasquez A, Moyerbrailean G, Land S, Djuric Z, Sun J, . Nutritional correlates of human oral microbiome. J Am Coll Nutr 2017;36:88–98. 27797671 10.1080/07315724.2016.1185386 PMC5477991

[17] Kim KA , Gu W, Lee IA, Joh EH, Kim DH. High fat diet-induced gut microbiota exacerbates inflammation and obesity in mice via the TLR4 signaling pathway. PLoS One 2012;7:e47713. 23091640 10.1371/journal.pone.0047713 PMC3473013

[18] Aune D , Norat T, Romundstad P, Vatten LJ. Whole grain and refined grain consumption and the risk of type 2 diabetes: a systematic review and dose-response meta-analysis of cohort studies. Eur J Epidemiol 2013;28:845–58. 24158434 10.1007/s10654-013-9852-5

[19] Borgi L , Rimm EB, Willett WC, Forman JP. Potato intake and incidence of hypertension: results from three prospective US cohort studies. BMJ 2016;353:i2351. 27189229 10.1136/bmj.i2351 PMC4870381

[20] Ludwig DS , Hu FB, Tappy L, Brand-Miller J. Dietary carbohydrates: role of quality and quantity in chronic disease. BMJ 2018;361:k2340. 29898880 10.1136/bmj.k2340 PMC5996878

[21] Livesey G , Taylor R, Hulshof T, Howlett J. Glycemic response and health—a systematic review and meta-analysis: relations between dietary glycemic properties and health outcomes. Am J Clin Nutr 2008;87:258S–68S. 18175766 10.1093/ajcn/87.1.258S

[22] Jayedi A , Soltani S, Jenkins D, Sievenpiper J, Shab-Bidar S. Dietary glycemic index, glycemic load, and chronic disease: an umbrella review of meta-analyses of prospective cohort studies. Crit Rev Food Sci Nutr 2020;62:2460–9. 33261511 10.1080/10408398.2020.1854168

[23] Shahdadian F , Saneei P, Milajerdi A, Esmaillzadeh A. Dietary glycemic index, glycemic load, and risk of mortality from all causes and cardiovascular diseases: a systematic review and dose-response meta-analysis of prospective cohort studies. Am J Clin Nutr 2019;110:921–37. 31187856 10.1093/ajcn/nqz061

[24] Negrini TC , Carlos IZ, Duque C, Caiaffa KS, Arthur RA. Interplay among the oral microbiome, oral cavity conditions, the host immune response, diabetes mellitus, and its associated-risk factors-an overview. Front Oral Health 2021;2:697428. 35048037 10.3389/froh.2021.697428 PMC8757730

[25] Galland L . Diet and inflammation. Nutr Clin Pract 2010;25:634–40. 21139128 10.1177/0884533610385703

[26] Sun L , Middleton DR, Wantuch PL, Ozdilek A, Avci FY. Carbohydrates as T-cell antigens with implications in health and disease. Glycobiology 2016;26:1029–40. 27236197 10.1093/glycob/cww062 PMC6086537

[27] Gnagnarella P , Gandini S, La Vecchia C, Maisonneuve P. Glycemic index, glycemic load, and cancer risk: a meta-analysis. Am J Clin Nutr 2008;87:1793–801. 18541570 10.1093/ajcn/87.6.1793

[28] Turati F , Galeone C, Gandini S, Augustin LS, Jenkins DJ, Pelucchi C, . High glycemic index and glycemic load are associated with moderately increased cancer risk. Mol Nutr Food Res 2015;59:1384–94. 25693843 10.1002/mnfr.201400594

[29] Qin H , Li G, Xu X, Zhang C, Zhong W, Xu S, . The role of oral microbiome in periodontitis under diabetes mellitus. J Oral Microbiol 2022;14:2078031. 35694215 10.1080/20002297.2022.2078031 PMC9176325

[30] Hayes RB , Reding D, Kopp W, Subar AF, Bhat N, Rothman N, . Etiologic and early marker studies in the prostate, lung, colorectal and ovarian (PLCO) cancer screening trial. Control Clin Trials 2000;21:349S–55S. 11189687 10.1016/s0197-2456(00)00101-x

[31] Calle EE , Rodriguez C, Jacobs EJ, Almon ML, Chao A, McCullough ML, . The American Cancer Society Cancer Prevention Study II Nutrition Cohort: rationale, study design, and baseline characteristics. Cancer 2002;94:2490–501. 12015775 10.1002/cncr.101970

[32] Jiao L , Flood A, Subar AF, Hollenbeck AR, Schatzkin A, Stolzenberg-Solomon R. Glycemic index, carbohydrates, glycemic load, and the risk of pancreatic cancer in a prospective cohort study. Cancer Epidemiol Biomarkers Prev 2009;18:1144–51. 19336549 10.1158/1055-9965.EPI-08-1135 PMC2687095

[33] Laaksonen DE , Toppinen LK, Juntunen KS, Autio K, Liukkonen KH, Poutanen KS, . Dietary carbohydrate modification enhances insulin secretion in persons with the metabolic syndrome. Am J Clin Nutr 2005;82:1218–27. 16332654 10.1093/ajcn/82.6.1218

[34] Okura Y , Urban LH, Mahoney DW, Jacobsen SJ, Rodeheffer RJ. Agreement between self-report questionnaires and medical record data was substantial for diabetes, hypertension, myocardial infarction and stroke but not for heart failure. J Clin Epidemiol 2004;57:1096–103. 15528061 10.1016/j.jclinepi.2004.04.005

[35] Shikany JM , Flood AP, Kitahara CM, Hsing AW, Meyer TE, Willcox BJ, . Dietary carbohydrate, glycemic index, glycemic load, and risk of prostate cancer in the Prostate, Lung, Colorectal, and Ovarian Cancer Screening Trial (PLCO) cohort. Cancer Causes Control 2011;22:995–1002. 21553078 10.1007/s10552-011-9772-1 PMC4470253

[36] Mongiovi JM , Freudenheim JL, Moysich KB, McCann SE. Glycemic index, glycemic load, and risk of ovarian cancer in the prostate, lung, colorectal and ovarian (PLCO) Cohort. J Nutr 2021;151:1597–608. 33693724 10.1093/jn/nxab011 PMC8169811

[37] Flood A , Peters U, Jenkins DJ, Chatterjee N, Subar AF, Church TR, . Carbohydrate, glycemic index, and glycemic load and colorectal adenomas in the Prostate, Lung, Colorectal, and Ovarian Screening Study. Am J Clin Nutr 2006;84:1184–92. 17093173 10.1093/ajcn/84.5.1184

[38] Flood A , Subar AF, Hull SG, Zimmerman TP, Jenkins DJ, Schatzkin A. Methodology for adding glycemic load values to the National Cancer Institute Diet History Questionnaire database. J Am Diet Assoc 2006;106:393–402. 16503230 10.1016/j.jada.2005.12.008

[39] Hu FB , Rimm E, Smith-Warner SA, Feskanich D, Stampfer MJ, Ascherio A, . Reproducibility and validity of dietary patterns assessed with a food-frequency questionnaire. Am J Clin Nutr 1999;69:243–9. 9989687 10.1093/ajcn/69.2.243

[40] Salmerón J , Manson JE, Stampfer MJ, Colditz GA, Wing AL, Willett WC. Dietary fiber, glycemic load, and risk of non-insulin-dependent diabetes mellitus in women. JAMA 1997;277:472–7. 9020271 10.1001/jama.1997.03540300040031

[41] Feigelson HS , Rodriguez C, Robertson AS, Jacobs EJ, Calle EE, Reid YA, . Determinants of DNA yield and quality from buccal cell samples collected with mouthwash. Cancer Epidemiol Biomarkers Prev 2001;10:1005–8. 11535555

[42] Fan X , Peters BA, Min D, Ahn J, Hayes RB. Comparison of the oral microbiome in mouthwash and whole saliva samples. PLoS One 2018;13:e0194729. 29641531 10.1371/journal.pone.0194729 PMC5894969

[43] Wu J , Lin I, Hayes RB, Ahn J. Comparison of DNA extraction methods for human oral microbiome research. Br J Med Med Res 2014;4:180–91.

[44] Caporaso JG , Kuczynski J, Stombaugh J, Bittinger K, Bushman FD, Costello EK, . QIIME allows analysis of high-throughput community sequencing data. Nat Methods 2010;7:335–6. 20383131 10.1038/nmeth.f.303 PMC3156573

[45] Chen T , Yu WH, Izard J, Baranova OV, Lakshmanan A, Dewhirst FE. The Human Oral Microbiome Database: a web accessible resource for investigating oral microbe taxonomic and genomic information. Database 2010;2010:baq013. 20624719 10.1093/database/baq013 PMC2911848

[46] Wu J , Peters BA, Dominianni C, Zhang Y, Pei Z, Yang L, . Cigarette smoking and the oral microbiome in a large study of American adults. ISME J 2016;10:2435–46. 27015003 10.1038/ismej.2016.37 PMC5030690

[47] Atkinson FS , Foster-Powell K, Brand-Miller JC. International tables of glycemic index and glycemic load values: 2008. Diabetes Care 2008;31:2281–3. 18835944 10.2337/dc08-1239 PMC2584181

[48] Oksanen J , Blanchet FG, Friendly M, Kindt R, Legendre P, McGlinn D, . vegan: Community Ecology Package R package version 2.5–7; 2020.

[49] McMurdie PJ , Holmes S. Phyloseq: an R package for reproducible interactive analysis and graphics of microbiome census data. PLoS One 2013;8:e61217. 23630581 10.1371/journal.pone.0061217 PMC3632530

[50] Love MI , Huber W, Anders S. Moderated estimation of fold change and dispersion for RNA-seq data with DESeq2. Genome Biol 2014;15:550. 25516281 10.1186/s13059-014-0550-8 PMC4302049

[51] Quinn TP , Erb I, Richardson MF, Crowley TM. Understanding sequencing data as compositions: an outlook and review. Bioinformatics 2018;34:2870–8. 29608657 10.1093/bioinformatics/bty175 PMC6084572

[52] Calgaro M , Romualdi C, Waldron L, Risso D, Vitulo N. Assessment of statistical methods from single cell, bulk RNA-seq, and metagenomics applied to microbiome data. Genome Biol 2020;21:191. 32746888 10.1186/s13059-020-02104-1 PMC7398076

[53] Asnicar F , Weingart G, Tickle TL, Huttenhower C, Segata N. Compact graphical representation of phylogenetic data and metadata with GraPhlAn. PeerJ 2015;3:e1029. 26157614 10.7717/peerj.1029 PMC4476132

[54] Humphrey LT , De Groote I, Morales J, Barton N, Collcutt S, Ramsey CB, . Earliest evidence for caries and exploitation of starchy plant foods in Pleistocene hunter-gatherers from Morocco. Proc Natl Acad Sci U S A 2014;111:954–9. 24395774 10.1073/pnas.1318176111 PMC3903197

[55] Baker JL , Edlund A. Exploiting the oral microbiome to prevent tooth decay: has evolution already provided the best tools? Front Microbiol 2019;9:3323. 30687294 10.3389/fmicb.2018.03323 PMC6338091

[56] Cross BW , Ruhl S. Glycan recognition at the saliva - oral microbiome interface. Cell Immunol 2018;333:19–33. 30274839 10.1016/j.cellimm.2018.08.008 PMC6296888

[57] Lamont RJ , Hajishengallis G. Polymicrobial synergy and dysbiosis in inflammatory disease. Trends Mol Med 2015;21:172–83. 25498392 10.1016/j.molmed.2014.11.004 PMC4352384

[58] Schwabe RF , Jobin C. The microbiome and cancer. Nat Rev Cancer 2013;13:800–12. 24132111 10.1038/nrc3610 PMC3986062

[59] von Frieling J , Fink C, Hamm J, Klischies K, Forster M, Bosch TCG, . Grow with the challenge – microbial effects on epithelial proliferation, carcinogenesis, and cancer therapy. Front Microbiol 2018;9:2020. 30294304 10.3389/fmicb.2018.02020 PMC6159313

[60] Graves DT , Correa JD, Silva TA. The oral microbiota is modified by systemic diseases. J Dent Res 2019;98:148–56. 30359170 10.1177/0022034518805739 PMC6761737

[61] Signat B , Roques C, Poulet P, Duffaut D. Fusobacterium nucleatum in periodontal health and disease. Curr Issues Mol Biol 2011;13:25–36. 21220789

[62] Gholizadeh P , Eslami H, Yousefi M, Asgharzadeh M, Aghazadeh M, Kafil HS. Role of oral microbiome on oral cancers, a review. Biomed Pharmacother 2016;84:552–8. 27693964 10.1016/j.biopha.2016.09.082

[63] Mitsuhashi K , Nosho K, Sukawa Y, Matsunaga Y, Ito M, Kurihara H, . Association of Fusobacterium species in pancreatic cancer tissues with molecular features and prognosis. Oncotarget 2015;6:7209–20. 25797243 10.18632/oncotarget.3109 PMC4466679

[64] McCoy AN , Araújo-Pérez F, Azcárate-Peril A, Yeh JJ, Sandler RS, Keku TO. Fusobacterium is associated with colorectal adenomas. PLoS One 2013;8:e53653. 23335968 10.1371/journal.pone.0053653 PMC3546075

[65] Aleksandar DK , Chun E, Robertson L, Jonathan NG, Carey AG, Michaud M, . Fusobacterium nucleatum potentiates intestinal tumorigenesis and modulates the tumor-immune microenvironment. Cell Host Microbe 2013;14:207–15. 23954159 10.1016/j.chom.2013.07.007 PMC3772512

[66] Sekizuka T , Ogasawara Y, Ohkusa T, Kuroda M. Characterization of Fusobacterium varium Fv113-g1 isolated from a patient with ulcerative colitis based on complete genome sequence and transcriptome analysis. PLoS One 2017;12:e0189319. 29216329 10.1371/journal.pone.0189319 PMC5720691

[67] Warren RL , Freeman DJ, Pleasance S, Watson P, Moore RA, Cochrane K, . Co-occurrence of anaerobic bacteria in colorectal carcinomas. Microbiome 2013;1:16. 24450771 10.1186/2049-2618-1-16 PMC3971631

[68] Han YW , Wang X. Mobile microbiome: oral bacteria in extra-oral infections and inflammation. J Dent Res 2013;92:485–91. 23625375 10.1177/0022034513487559 PMC3654760

[69] Bullman S , Pedamallu CS, Sicinska E, Clancy TE, Zhang X, Cai D, . Analysis of Fusobacterium persistence and antibiotic response in colorectal cancer. Science 2017;358:1443–8. 29170280 10.1126/science.aal5240 PMC5823247

[70] Lu S , Wang J, Chitsaz F, Derbyshire MK, Geer RC, Gonzales NR, . CDD/SPARCLE: the conserved domain database in 2020. Nucleic Acids Res 2020;48:D265–8. 31777944 10.1093/nar/gkz991 PMC6943070

[71] Liu L , Tabung FK, Zhang X, Nowak JA, Qian ZR, Hamada T, . Diets that promote colon inflammation associate with risk of colorectal carcinomas that contain fusobacterium nucleatum. Clin Gastroenterol Hepatol 2018;16:1622–31. 29702299 10.1016/j.cgh.2018.04.030 PMC6151288

[72] Ranjbar M , Salehi R, Javanmard SH, Rafiee L, Faraji H, jafarpor S, . The dysbiosis signature of Fusobacterium nucleatum in colorectal cancer-cause or consequences? A systematic review. Cancer Cell Int 2021;21:194. 33823861 10.1186/s12935-021-01886-z PMC8025348

[73] Long J , Cai Q, Steinwandel M, Hargreaves MK, Bordenstein SR, Blot WJ, . Association of oral microbiome with type 2 diabetes risk. J Periodontal Res 2017;52:636–43. 28177125 10.1111/jre.12432 PMC5403709

[74] Kim SR , Lee YH, Lee SG, Kang ES, Cha BS, Kim JH, . Urinary N-acetyl-beta-D-glucosaminidase, an early marker of diabetic kidney disease, might reflect glucose excursion in patients with type 2 diabetes. Medicine 2016;95:e4114. 27399115 10.1097/MD.0000000000004114 PMC5058844

[75] Said HS , Suda W, Nakagome S, Chinen H, Oshima K, Kim S, . Dysbiosis of salivary microbiota in inflammatory bowel disease and its association with oral immunological biomarkers. DNA Res 2014;21:15–25. 24013298 10.1093/dnares/dst037 PMC3925391

[76] McDonald C , Inohara N, Nuñez G. Peptidoglycan signaling in innate immunity and inflammatory disease. J Biol Chem 2005;280:20177–80. 15802263 10.1074/jbc.R500001200

[77] Radwan S , Gilfillan D, Eklund B, Radwan HM, El Menofy NG, Lee J, . A comparative study of the gut microbiome in Egyptian patients with type I and type II diabetes. PLoS One 2020;15:e0238764. 32903276 10.1371/journal.pone.0238764 PMC7480833

[78] Millen AE , Dahhan R, Freudenheim JL, Hovey KM, Li L, McSkimming DI, . Dietary carbohydrate intake is associated with the subgingival plaque oral microbiome abundance and diversity in a cohort of postmenopausal women. Sci Rep 2022;12:2643. 35173205 10.1038/s41598-022-06421-2 PMC8850494

