# Supplementary material for: Elevated Dietary Carbohydrate and Glycemic Intake Associate with an Altered Oral Microbial Ecosystem in Two Large U.S. Cohorts
Source: Cancer Res Commun. 2022 Dec 5;2(12):1558–68. doi: 10.1158/2767-9764.CRC-22-0323 (PMC9770587; doi:10.1158/2767-9764.CRC-22-0323)

Supplementary Data

Figures

Supplementary Figure S1. Population flow diagram depicting initial study cohorts, exclusion criteria, and final study population for analysis. NCI PLCO: National Cancer Institute Prostate, Lung, Colorectal, and Ovarian Cancer Screening Trial; ACS CPS-II: American Cancer Society Cancer Prevention Study II; H&N: Head and Neck cancer; FFQ: Food Frequency Questionnaire.

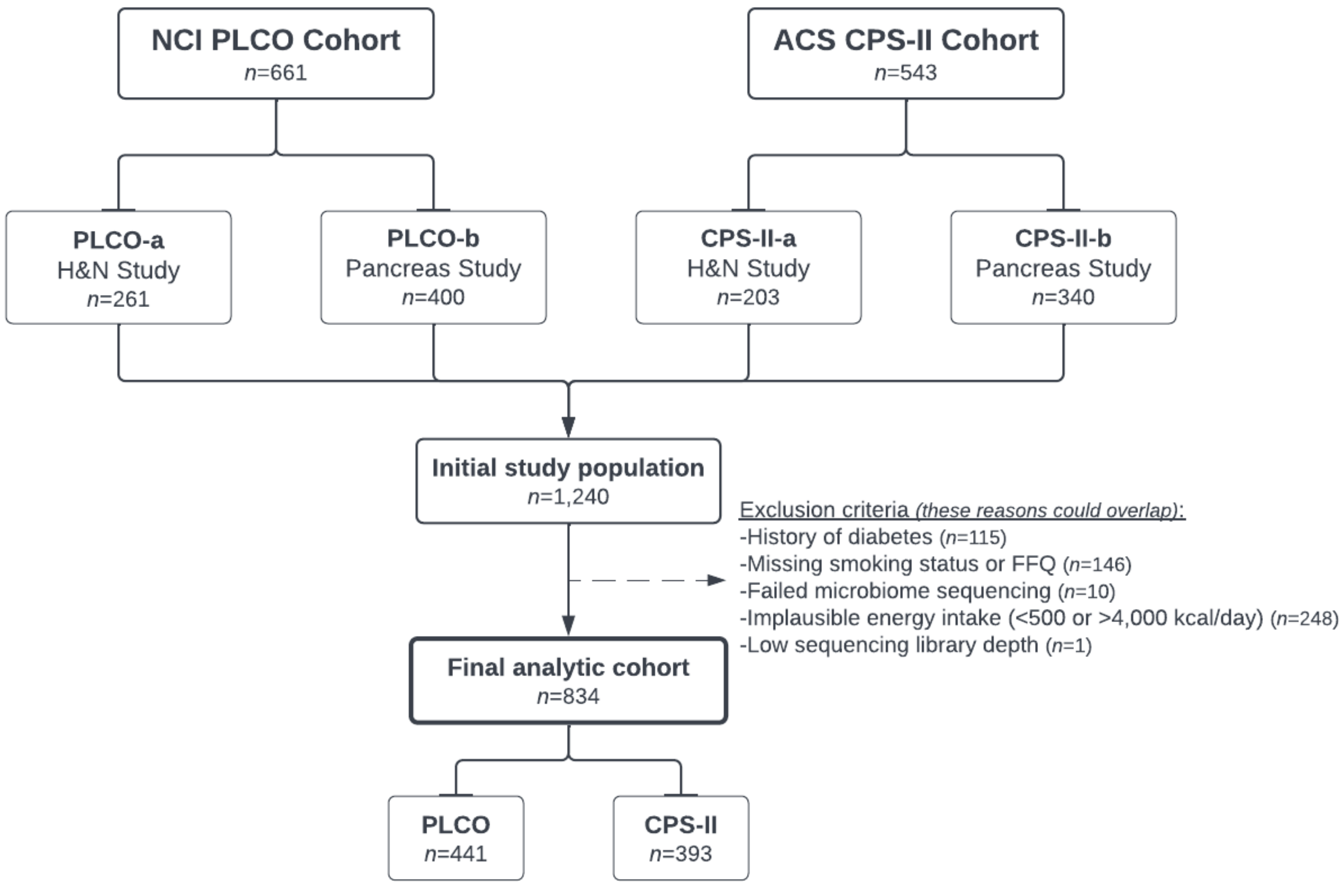

Supplement: Figure S1 — Population flow diagram [file crc-22-0323-s01.pdf]
