# Supplementary material for: Elevated Dietary Carbohydrate and Glycemic Intake Associate with an Altered Oral Microbial Ecosystem in Two Large U.S. Cohorts
Source: Cancer Res Commun. 2022 Dec 5;2(12):1558–68. doi: 10.1158/2767-9764.CRC-22-0323 (PMC9770587; doi:10.1158/2767-9764.CRC-22-0323)

Supplementary Figure S2. Pearson Correlation test and simple linear regression line for the association between carbohydrates and Glycemic Index (GI) in the PLCO and CPS-II cohorts (n=834). Overall there is modest but significant correlation between the two measures (Pearson Correlation Coefficient=0.12, p=6.6E-04). Points indicate individual subject carbohydrate and GI intake; solid black line and gray bands are the linear regression line and corresponding 95% confidence interval, respectively.

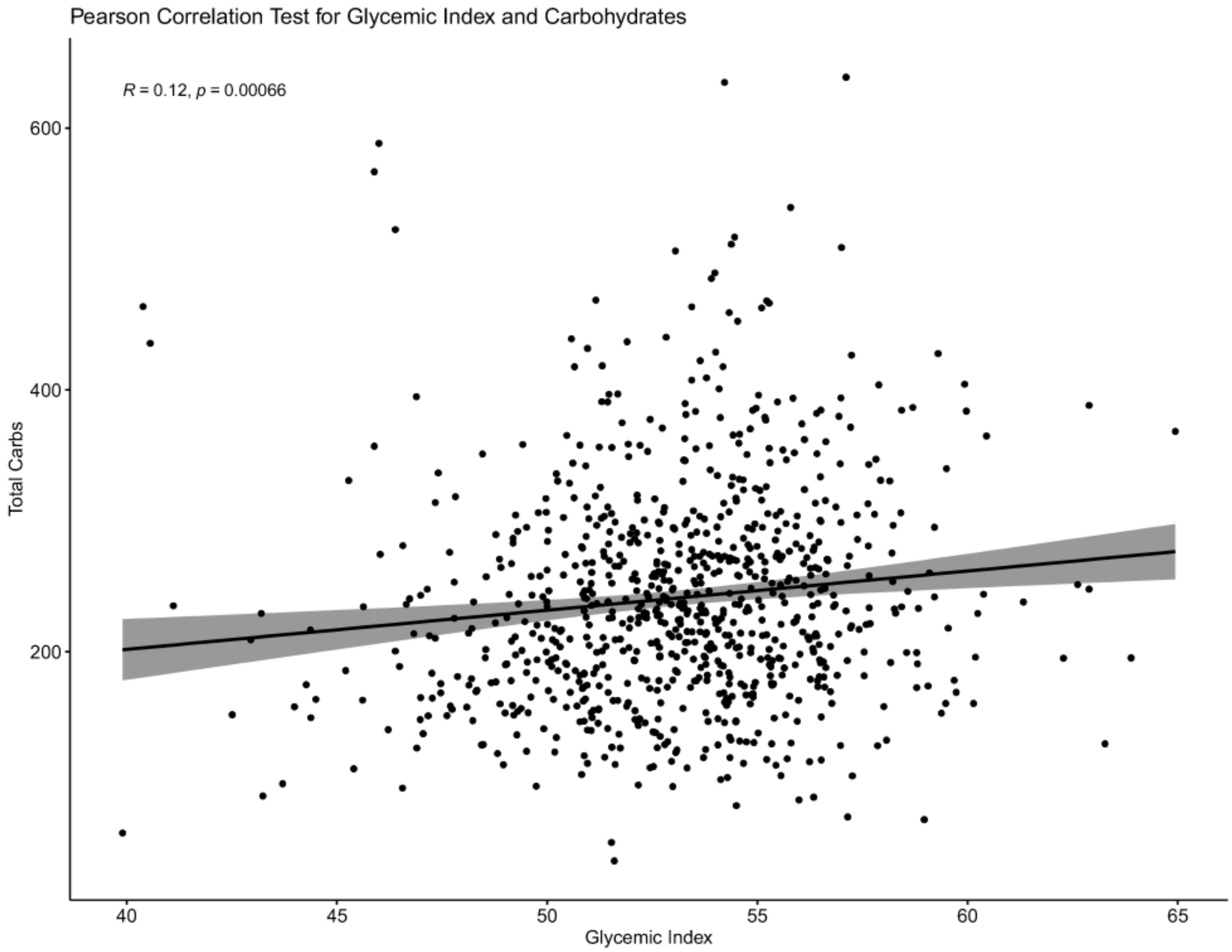

Supplement: Figure S2 — Carbohydrate and GI Pearson correlation [file crc-22-0323-s02.pdf]
