# Supplementary material for: Elevated Dietary Carbohydrate and Glycemic Intake Associate with an Altered Oral Microbial Ecosystem in Two Large U.S. Cohorts
Source: Cancer Res Commun. 2022 Dec 5;2(12):1558–68. doi: 10.1158/2767-9764.CRC-22-0323 (PMC9770587; doi:10.1158/2767-9764.CRC-22-0323)
Supplement: Table S1 — Carbohydrate and GI Alpha diversity [file crc-22-0323-s03.pdf]

Tables

Supplementary Table S1. Association of α-diversity metrics with daily carbohydrate and Glycemic Index (GI) as categorical (quintiles) and continuous variables, PLCO and ACS cohorts (n=834)

	Categorical β (95% CI)[a]						Continuous[b] β (95% CI)[a]	
	Q1	Q2	Q3	Q4	Q5	*P*-trend		*P*
**Carbohydrate**								
Richness	*Ref.*	2.59 (-4.86, 10.0)	6.79 (-0.90, 14.5)	2.33 (-5.76, 10.4)	6.05 (-3.19, 15.3)	0.27	0.03 (-3.15, 3.20)	0.99
Shannon index	*Ref.*	-0.02 (-0.16, 0.12)	0.14 (-0.01, 0.29)	0.04 (-0.12, 0.19)	0.17 (-0.01, 0.35)	*0.06*	0.03 (-0.03, 0.09)	0.35
Evenness	*Ref.*	-0.01 (-0.02, 0.01)	0.01 (-0.01, 0.03)	0.002 (-0.02, 0.02)	0.02 (-0.002, 0.04)	*0.07*	0.004 (-0.003, 0.01)	0.25
**Glycemic Index**								
Richness	*Ref.*	-3.69 (-11.1, 3.76)	1.56 (-5.95, 9.06)	-5.78 (-13.3, 1.72)	-4.88 (-12.5, 2.74)	0.17	-2.01 (-4.48, 0.45)	0.11
Shannon index	*Ref.*	-0.14 (-0.28, 0.01)	0.02 (-0.13, 0.16)	-0.09 (-0.23, 0.06)	-0.13 (-0.28, 0.02)	0.22	-0.03 (-0.08, 0.02)	0.19
Evenness	*Ref.*	-0.02 (-0.03, 0)	0 (-0.02, 0.02)	-0.01 (-0.02, 0.01)	-0.01 (-0.03, 0.004)	0.43	-0.002 (-0.007, 0.003)	0.48

[a]Regression parameters and *P*-values are from linear regression models with specified α-diversity metric (richness, Shannon diversity index, community evenness, averaged over 100 iterations of rarefied OTU table at 3,000 sequence reads/sample) as the outcome. All models were adjusted for age, sex, study (PLCOa, PLCOb, CPS-IIa, CPS-IIb), current smoking, BMI (kg/m2), energy intake (kcal/day), and alcohol intake (grams/day).

[b]Continuous values scaled by 1 S.D. unit increase
