# Supplementary material for: Elevated Dietary Carbohydrate and Glycemic Intake Associate with an Altered Oral Microbial Ecosystem in Two Large U.S. Cohorts
Source: Cancer Res Commun. 2022 Dec 5;2(12):1558–68. doi: 10.1158/2767-9764.CRC-22-0323 (PMC9770587; doi:10.1158/2767-9764.CRC-22-0323)
Supplement: Table S2 — Carbohydrate and GI Beta diversity [file crc-22-0323-s04.pdf]

Supplementary Table S2. Association of β-diversity metrics with daily carbohydrate and Glycemic Index (GI) and as categorical (quintiles) and continuous variables, PLCO and ACS cohorts (n=834)

	**Categorical**						**Continuous**
	P-value[a]						*P*-value[a]
	**Q1**	**Q2**	**Q3**	**Q4**	**Q5**	***P*-trend**	**Per day**
**Carbohydrate**	*Ref.*	0.77	0.71	0.80	0.56	0.71	0.38
**Glycemic Index**	*Ref.*	0.23	0.78	0.30	0.23	0.33	0.26

[a]*P*-values are from PERMANOVA of weighted UniFrac distance adjusted for age, sex, study (PLCOa, PLCOb, CPS-IIa, CPS-IIb), current smoking, BMI (kg/m2), energy intake (kcal/day), and alcohol intake (grams/day).
