# Supplementary material for: Elevated Dietary Carbohydrate and Glycemic Intake Associate with an Altered Oral Microbial Ecosystem in Two Large U.S. Cohorts
Source: Cancer Res Commun. 2022 Dec 5;2(12):1558–68. doi: 10.1158/2767-9764.CRC-22-0323 (PMC9770587; doi:10.1158/2767-9764.CRC-22-0323)
Supplement: Table S3 — Sensitivity analyses, alpha diversity [file crc-22-0323-s05.pdf]

Supplementary Table S3(a). Association of α-diversity metrics with daily carbohydrates and Glycemic Index (GI) as categorical (quintiles) and continuous variables, stratified by BMI (normal: < kg/m^2, n=328; elevated: ≥25-< kg/m^2, n=358; high: ≥ kg/m^2, n=148)

BMI Normal: <25kg/m^2

	Categorical						Continuous	
	β (95% CI)[a,b]						β (95% CI)[a,b]	
	Quintile 1	Quintile 2	Quintile 3	Quintile 4	Quintile 5	*P*-trend[a]	Per Day	*P*[a]
**Carbohydrates**								
Richness	*Ref.*	0.45 (-11.7, 12.6)	7.21 (-4.68, 19.1)	10.4 (-2.7, 23.4)	7.42 (-7.26, 22.1)	0.11	0.02 (-0.04, 0.08)	0.55
Shannon Index	*Ref.*	-0.01 (-0.23, 0.22)	0.16 (-0.06, 0.39)	0.14 (-0.10, 0.39)	0.20 (-0.08, 0.47)	0.07	0 (-0.001, 0.001)	0.47
Evenness	*Ref.*	-0.002 (-0.03, 0.02)	0.01 (-0.01, 0.04)	0.01 (-0.02, 0.03)	0.02 (-0.01, 0.05)	0.13	0 (0, 0)	0.53
**Glycemic Index**								
Richness	*Ref.*	0.70 (-11.0, 12.4)	4.76 (-6.62, 16.1)	-4.23 (-16.3, 7.82)	-3.91 (-16.0, 8.21)	0.38	-0.55 (-1.76, 0.65)	0.37
Shannon Index	*Ref.*	-0.14 (-0.36, 0.07)	0.02 (-0.20, 0.23)	-0.20 (-0.43, 0.02)	-0.21 (-0.43, 0.02)	0.07	-0.14 (-0.36, 0.07)	0.20
Evenness	*Ref.*	-0.02 (-0.05, 0)	-0.003 (-0.03, 0.02)	**-0.03 (-0.05, -0.002)***	**-0.03 (-0.05, -0.002)***	**0.05***	0 (0, 0)	0.53

BMI Elevated: ≥25-<30 kg/m^2

	Categorical						Continuous	
	β (95% CI)[a,b]						β (95% CI)[a,b]	
	Quintile 1	Quintile 2	Quintile 3	Quintile 4	Quintile 5	*P*-trend[a]	Per Day	*P*
**Carbohydrates**								
Richness	*Ref.*	-6.12 (-18.0, 5.81)	7.56 (-5.30, 20.4)	1.71 (-10.7, 14.2)	6.35 (-7.70, 20.4)	0.21	0.02 (-0.04, 0.08)	0.55
Shannon Index	*Ref.*	-0.19 (-0.44, 0.05)	0.1 (-0.16, 0.36)	0.02 (-0.23, 0.28)	0.14 (-0.15, 0.43)	0.14	0.001 (-0.001, 0.002)	0.27
Evenness	*Ref.*	-0.02 (-0.05, 0.01)	0.01 (-0.02, 0.04)	0.002 (-0.03, 0.03)	0.02 (-0.02, 0.05)	0.16	0 (0, 0)	0.21
**Glycemic Index**								
Richness	*Ref.*	-5.62 (-18.2, 6.93)	3.87 (-8.71, 16.5)	-5.54 (-18.0, 6.96)	-5.47 (-17.8, 6.89)	0.44	-0.59 (-1.79, 0.62)	0.34
Shannon Index	*Ref.*	-0.07 (-0.32, 0.19)	0.04 (-0.22, 0.29)	-0.04 (-0.29, 0.22)	-0.11 (-0.37, 0.14)	0.42	-0.066 (-0.323, 0.191)	0.62
Evenness	*Ref.*	-0.004 (-0.03, 0.02)	-0.001 (-0.03, 0.03)	0 (-0.03, 0.03)	-0.01 (-0.04, 0.02)	0.63	0 (0, 0)	0.21

BMI High: ≥30 kg/m²

	Categorical						Continuous	
	β (95% CI)[a,b]						β (95% CI)[a,b]	
	Quintile 1	Quintile 2	Quintile 3	Quintile 4	Quintile 5	*P*-trend[a]	Per Day	*P*
**Carbohydrates**								
Richness	*Ref.*	**28.6 (11.5, 45.7)***	13.7 (-3.13, 30.6)	1.06 (-16.6, 18.7)	**21.1 (0.15, 42.1)***	0.74	0.03 (-0.05, 0.12)	0.48
Shannon Index	*Ref.*	**0.40 (0.06, 0.74)***	**0.36 (0.02, 0.69)***	0.12 (-0.23, 0.47)	**0.51 (0.10, 0.93)***	0.17	0.001 (0, 0.003)	0.17
Evenness	*Ref.*	0.02 (-0.02, 0.06)	0.03 (-0.01, 0.07)	0.01 (-0.03, 0.05)	0.05 (-0.002, 0.09)	0.16	0 (0, 0)	0.20
**Glycemic Index**								
Richness	*Ref.*	-7.46 (-24.5, 9.56)	-9.35 (-28.5, 9.80)	-9.27 (-26.5, 7.97)	-0.40 (-18.3, 17.5)	0.92	-0.45 (-2.12, 1.22)	0.60
Shannon Index	*Ref.*	-0.06 (-0.39, 0.27)	0.18 (-0.19, 0.55)	0.16 (-0.17, 0.50)	0.22 (-0.13, 0.56)	0.08	-0.06 (-0.39, 0.27)	0.73
Evenness	*Ref.*	0.003 (-0.03, 0.04)	0.04 (-0.001, 0.08)	**0.04 (0, 0.07)**	0.03 (-0.003, 0.07)	**0.01***	0 (0, 0)	0.20

[a]Regression parameters and *P*-values are from linear regression models with specified α-diversity metric (richness, Shannon diversity index, community evenness, averaged over 100 iterations of rarefied OTU table at 3,000 sequence reads/sample) as the outcome. All models were adjusted for age, sex, study (PLCOa, PLCOb, CPS-IIa, CPS-IIb), current smoking, energy intake (kcal/day), and alcohol intake (grams/day).

[b]One star (*) indicates *P*-value < 0.05 from the linear regression model.

Supplementary Table S3(b). Association of α-diversity metrics with daily carbohydrates and Glycemic Index (GI) as categorical (quintiles) and continuous variables, stratified by cohort (PLCO, n=441, CPS-II, n=393)

PLCO

	Categorical						Continuous	
	β (95% CI)[a,b]						β (95% CI)[a,b]	
	Quintile 1	Quintile 2	Quintile 3	Quintile 4	Quintile 5	*P*-trend[a]	Per Day	*P*
**Total Carbohydrates**								
Richness	*Ref.*	1.35 (-8.45, 11.1)	5.42 (-4.52, 15.4)	-0.51 (-10.7, 9.73)	0.17 (-10.9, 11.3)	0.83	-0.004 (-0.05, 0.04)	0.83
Shannon Index	*Ref.*	0.03 (-0.18, 0.23)	0.10 (-0.11, 0.30)	0.07 (-0.14, 0.29)	0.15 (-0.08, 0.38)	0.20	0 (0, 0.001)	0.32
Evenness	*Ref.*	0.002 (-0.02, 0.03)	0.01 (-0.02, 0.03)	0.01 (-0.02, 0.03)	0.02 (-0.01, 0.05)	0.13	0 (0, 0)	0.21
**Glycemic Index**								
Richness	*Ref.*	-9.33 (-19.2, 0.53)	3.40 (-6.56, 13.4)	-7.46 (-17.5, 2.60)	**-10.4 (-20.6, -0.21)***	0.11	-0.89 (-1.89, 0.11)	0.08
Shannon Index	*Ref.*	**-0.25 (-0.45, -0.04)***	-0.01 (-0.22, 0.20)	-0.11 (-0.32, 0.10)	**-0.30 (-0.51, -0.09)***	**0.05***	**-0.25 (-0.45, -0.04)**	**0.02***
Evenness	*Ref.*	**-0.02 (-0.05, -0.001)***	-0.01 (-0.03, 0.02)	-0.01 (-0.03, 0.02)	**-0.03 (-0.05, -0.01)***	0.13	-0.002 (-0.004, 0.001)	0.14

CPS-II

	Categorical						Continuous	
	β (95% CI)[a,b]						β (95% CI)[a,b]	
	Quintile 1	Quintile 2	Quintile 3	Quintile 4	Quintile 5	*P*-trend[a]	Per Day	*P*
**Total Carbohydrates**								
Richness	*Ref.*	-0.85 (-12.5, 10.8)	1.52 (-11.4, 14.5)	-2.53 (-17.6, 12.5)	-2.70 (-22.8, 17.4)	0.89	-0.11 (-0.22, 0.004)	0.06
Shannon Index	*Ref.*	-0.09 (-0.29, 0.11)	0.05 (-0.17, 0.27)	-0.12 (-0.37, 0.14)	-0.17 (-0.51, 0.18)	0.56	**-0.003 (-0.004, -0.001)***	**0.01***
Evenness	*Ref.*	-0.01 (-0.03, 0.01)	0.004 (-0.02, 0.03)	-0.01 (-0.04, 0.01)	-0.02 (-0.06, 0.01)	0.51	**0 (0, 0)***	**0.01***
**Glycemic Index**								
Richness	*Ref.*	1.05 (-10.2, 12.3)	-2.97 (-14.2, 8.26)	-5.22 (-16.4, 5.95)	-5.57 (-16.9, 5.76)	0.20	-0.79 (-1.91, 0.33)	0.17
Shannon Index	*Ref.*	-0.03 (-0.23, 0.16)	-0.02 (-0.21, 0.18)	-0.08 (-0.28, 0.11)	-0.02 (-0.22, 0.17)	0.61	-0.01 (-0.03, 0.01)	0.35
Evenness	*Ref.*	-0.01 (-0.03, 0.01)	0 (-0.02, 0.02)	-0.01 (-0.03, 0.01)	0.004 (-0.02, 0.02)	0.82	0 (-0.002, 0.002)	0.81

[a]Regression parameters and *P*-values are from linear regression models with specified α-diversity metric (richness, Shannon diversity index, community evenness, averaged over 100 iterations of rarefied OTU table at 3,000 sequence reads/sample) as the outcome. All models were adjusted for age, sex, current smoking, BMI (mg/kg2), energy intake (kcal/day), and alcohol intake (grams/day).

[b]One star (*) indicates *P*-value < 0.05 from the linear regression model.

Supplementary Table S3(c). Association of α-diversity metrics with daily carbohydrate and Glycemic Index (GI) as categorical (quintiles) and continuous variables, stratified by sex (male, n=528 and female, n=306)

Male

	Categorical						Continuous	
	β (95% CI)[a,b]						β (95% CI)[a,b]	
	Quintile 1	Quintile 2	Quintile 3	Quintile 4	Quintile 5	*P*-trend[a]	Per Day	*P*
**Carbohydrates**								
Richness	*Ref.*	4.47 (-6.54, 15.5)	7.38 (-3.25, 18.0)	3.44 (-7.39, 14.3)	4.77 (-7.42, 17.0)	0.60	-0.02 (-0.07, 0.03)	0.41
Shannon Index	*Ref.*	0.04 (-0.17, 0.25)	0.13 (-0.07, 0.33)	0.05 (-0.15, 0.26)	0.14 (-0.09, 0.37)	0.29	0 (-0.001, 0.001)	0.78
Evenness	*Ref.*	0 (-0.02, 0.02)	0.01 (-0.01, 0.03)	0.002 (-0.02, 0.02)	0.01 (-0.01, 0.04)	0.31	0 (0, 0)	0.99
**Glycemic Index**								
Richness	*Ref.*	-4.24 (-14.4, 5.96)	1.80 (-8.43, 12.0)	-9.04 (-19.5, 1.42)	-4.54 (-14.8, 5.77)	0.25	-0.71 (-1.67, 0.25)	0.15
Shannon Index	*Ref.*	-0.13 (-0.32, 0.06)	0.03 (-0.16, 0.23)	-0.04 (-0.24, 0.16)	-0.04 (-0.23, 0.16)	0.83	-0.13 (-0.32, 0.06)	0.18
Evenness	*Ref.*	-0.01 (-0.03, 0.01)	0.002 (-0.02, 0.02)	0.01 (-0.02, 0.03)	0 (-0.02, 0.02)	0.30	0 (0, 0)	0.99

Female

	Categorical						Continuous	
	β (95% CI)[a,b]						β (95% CI)[a,b]	
	Quintile 1	Quintile 2	Quintile 3	Quintile 4	Quintile 5	*P*-trend[a]	Per Day	*P*
**Carbohydrates**								
Richness	*Ref.*	2.23 (-8.43, 12.9)	1.87 (-8.77, 12.5)	0.19 (-10.1, 10.4)	-1.42 (-12.5, 9.70)	0.77	-0.31 (-1.41, 0.79)	0.58
Shannon Index	*Ref.*	-0.06 (-0.28, 0.16)	0.03 (-0.19, 0.25)	-0.11 (-0.32, 0.10)	**-0.25 (-0.48, -0.03)***	**0.04***	-0.06 (-0.28, 0.16)	0.61
Evenness	*Ref.*	-0.01 (-0.04, 0.01)	0.002 (-0.02, 0.03)	-0.02 (-0.04, 0.01)	**-0.03 (-0.06, -0.01)***	**0.02***	0 (0, 0)	0.09
**Glycemic Index**								
Richness	*Ref.*	-0.78 (-10.3, 8.79)	1.89 (-9.08, 12.9)	-7.05 (-19.7, 5.59)	1.61 (-13.8, 17.0)	0.77	-0.01 (-0.08, 0.05)	0.70
Shannon Index	*Ref.*	-0.09 (-0.28, 0.11)	0.15 (-0.08, 0.37)	-0.02 (-0.28, 0.24)	0.18 (-0.14, 0.5)	0.29	0.001 (-0.001, 0.002)	0.25
Evenness	*Ref.*	-0.01 (-0.03, 0.01)	0.02 (-0.01, 0.04)	0.004 (-0.03, 0.03)	0.03 (-0.01, 0.06)	0.12	0 (0, 0)	0.09

[a]Regression parameters and *P*-values are from linear regression models with specified α-diversity metric (richness, Shannon diversity index, community evenness, averaged over 100 iterations of rarefied OTU table at 3,000 sequence reads/sample) as the outcome. All models were adjusted for age, study (PLCOa, PLCOb, CPS-IIa, CPS-IIb), current smoking, BMI (mg/kg2), energy intake (kcal/day), and alcohol intake (grams/day).

[b]One star (*) indicates *P*-value < 0.05 from the linear regression model.

Supplementary Table S3(d). Association of α-diversity metrics with daily carbohydrate and Glycemic Index (GI) as categorical (quintiles) and continuous variables in full dataset (PLCO and CPS-II cohorts) including diabetic subjects (n=938)

Full Dataset (including diabetic subjects)

	Categorical						Continuous	
	β (95% CI)[a,b]						β (95% CI)[a,b]	
	Quintile 1	Quintile 2	Quintile 3	Quintile 4	Quintile 5	*P*-trend[a]	Per Day	*P*
**Carbohydrates**								
Richness	*Ref.*	2.24 (-4.85, 9.33)	5.41 (-1.98, 12.8)	4.06 (-3.66, 11.8)	7.27 (-1.58, 16.1)	0.12	0.54 (-2.48, 3.56)	0.73
Shannon Index	*Ref.*	-0.01 (-0.14, 0.13)	0.12 (-0.02, 0.26)	0.08 (-0.07, 0.23)	**0.20 (0.03, 0.37)**	**0.02***	0.04 (-0.02, 0.1)	0.15
Evenness	*Ref.*	-0.003 (-0.02, 0.01)	0.01 (-0.01, 0.02)	0.01 (-0.01, 0.02)	**0.02 (0.002, 0.04)**	**0.02***	0.01 (-0.001, 0.01)	0.09
**Glycemic Index**								
Richness	*Ref.*	-1.04 (-8.14, 6.06)	2.83 (-4.29, 9.96)	-1.15 (-8.29, 6.00)	-1.32 (-8.55, 5.92)	0.73	-0.47 (-2.81, 1.87)	0.69
Shannon Index	*Ref.*	-0.06 (-0.20, 0.08)	0.05 (-0.09, 0.19)	0.02 (-0.12, 0.15)	-0.05 (-0.19, 0.09)	0.86	-0.002 (-0.05, 0.04)	0.94
Evenness	*Ref.*	-0.01 (-0.02, 0.01)	0.003 (-0.01, 0.02)	0.003 (-0.01, 0.02)	-0.01 (-0.02, 0.01)	0.99	0.001 (-0.004, 0.01)	0.80

[a]Regression parameters and *P*-values are from linear regression models with specified α-diversity metric (richness, Shannon diversity index, community evenness, averaged over 100 iterations of rarefied OTU table at 3,000 sequence reads/sample) as the outcome. All models were adjusted for age, study (PLCOa, PLCOb, CPS-IIa, CPS-IIb), current smoking, BMI (mg/kg2), energy intake (kcal/day), and alcohol intake (grams/day).

[b]One star (*) indicates *P*-value < 0.05 from the linear regression model.

Supplementary Table S3(e). Association of α-diversity metrics with daily carbohydrate and Glycemic Index (GI) as categorical (quintiles) and continuous variables when restricting to PLCO and CPS-II cohort controls (n=543)

PLCO and CPS-II Controls

	Categorical						Continuous	
	β (95% CI)[a,b]						β (95% CI)[a,b]	
	Quintile 1	Quintile 2	Quintile 3	Quintile 4	Quintile 5	*P*-trend[a]	Per Day	*P*
**Carbohydrates**								
Richness	*Ref.*	0.73 (-8.42, 9.88)	6.42 (-3.01, 15.8)	7.48 (-2.46, 17.4)	8.83 (-2.56, 20.2)	0.06	1.37 (-2.56, 5.30)	0.50
Shannon Index	*Ref.*	-0.08 (-0.25, 0.10)	0.07 (-0.11, 0.25)	0.05 (-0.14, 0.24)	0.21 (-0.01, 0.42)	**0.04***	0.05 (-0.03, 0.12)	0.22
Evenness	*Ref.*	-0.01 (-0.03, 0.01)	0.002 (-0.02, 0.02)	-0.002 (-0.02, 0.02)	0.02 (-0.004, 0.04)	0.08	0.01 (-0.003, 0.01)	0.22
**Glycemic Index**								
Richness	*Ref.*	-1.79 (-10.8, 7.25)	7.90 (-1.14, 16.9)	-1.07 (-10.1, 7.98)	-0.10 (-9.26, 9.06)	0.99	-0.11 (-3.10, 2.88)	0.94
Shannon Index	*Ref.*	-0.04 (-0.21, 0.13)	**0.20 (0.02, 0.37)**	0.10 (-0.07, 0.27)	0.08 (-0.10, 0.25)	0.16	0.04 (-0.02, 0.09)	0.23
Evenness	*Ref.*	-0.004 (-0.02, 0.01)	**0.02 (0.001, 0.04)**	0.02 (-0.003, 0.03)	0.01 (-0.01, 0.03)	0.06	0.01 (-0.001, 0.01)	0.10

[a]Regression parameters and *P*-values are from linear regression models with specified α-diversity metric (richness, Shannon diversity index, community evenness, averaged over 100 iterations of rarefied OTU table at 3,000 sequence reads/sample) as the outcome. All models were adjusted for age, study (PLCOa, PLCOb, CPS-IIa, CPS-IIb), current smoking, BMI (mg/kg2), energy intake (kcal/day), and alcohol intake (grams/day).

[b]One star (*) indicates *P*-value < 0.05 from the linear regression model.

Supplementary Table S3(f). Association of α-diversity metrics with daily carbohydrates as percent of calories as a categorical (quintiles) and continuous variable (n=834)

	Categorical						Continuous	
	β (95% CI)[a,b]						β (95% CI)[a,b]	
	Quintile 1	Quintile 2	Quintile 3	Quintile 4	Quintile 5	*P*-trend[a]	Per Day	*P*
**Carbohydrate as percent of calories**								
Richness	*Ref.*	6.75 (-1.03, 14.5)	**13.1 (3.92, 22.3)**	4.57 (-7.00, 16.1)	-0.32 (-12.4, 11.8)	0.81	-0.03 (-0.31, 0.25)	0.83
Shannon Index	*Ref.*	**0.16 (0.01, 0.31)**	**0.30 (0.12, 0.47)**	0.15 (-0.07, 0.38)	0.08 (-0.15, 0.32)	0.61	0.001 (-0.004, 0.01)	0.68
Evenness	*Ref.*	**0.02 (0, 0.03)**	**0.03 (0.01, 0.05)**	0.02 (-0.01, 0.04)	0.01 (-0.01, 0.04)	0.41	0 (0, 0.001)	0.50

[a]Regression parameters and *P*-values are from linear regression models with specified α-diversity metric (richness, Shannon diversity index, community evenness, averaged over 100 iterations of rarefied OTU table at 3,000 sequence reads/sample) as the outcome. All models were adjusted for age, study (PLCOa, PLCOb, CPS-IIa, CPS-IIb), current smoking, BMI (mg/kg2), and alcohol intake (grams/day).

[b]One star (*) indicates *P*-value < 0.05 from the linear regression model.

Supplementary Table S3(g). Association of α-diversity metrics with daily Glycemic Load (GL), Sucrose, and Fiber intake as categorical (quintiles) and continuous variables (n=834)

	**Categorical**						**Continuous**	
	β (95% CI)[a,b]						β (95% CI)[a,b]	
	Quintile 1	Quintile 2	Quintile 3	Quintile 4	Quintile 5	*P*-trend[a]	Per Day	*P*
**Glycemic Load**								
Richness	*Ref.*	1.17 (-6.37, 8.71)	0.47 (-7.41, 8.34)	3.00 (-5.40, 11.4)	4.10 (-5.74, 14.0)	0.40	0.03 (-0.05, 0.10)	0.49
Shannon Index	*Ref.*	-0.002 (-0.15, 0.14)	0.05 (-0.10, 0.20)	0.08 (-0.07, 0.24)	0.13 (-0.06, 0.32)	0.14	0.001 (0, 0.003)	0.11
Evenness	*Ref.*	-0.003 (-0.018, 0.013)	0.01 (-0.01, 0.02)	0.01 (-0.01, 0.02)	0.01 (-0.01, 0.03)	0.17	0 (0, 0)	0.10
**Sucrose**								
Richness	*Ref.*	-0.61 (-8.18, 6.96)	-3.25 (-11.3, 4.84)	-1.05 (-9.88, 7.79)	-10.7 (-21.8, 0.49)	0.18	-0.20 (-0.40, 0.01)	0.06
Shannon Index	*Ref.*	-0.05 (-0.20, 0.10)	-0.15 (-0.30, 0.01)	-0.05 (-0.22, 0.12)	-0.16 (-0.37, 0.06)	0.25	-0.002 (-0.01, 0.002)	0.27
Evenness	*Ref.*	-0.01 (-0.02, 0.01)	**-0.02 (-0.04, -0.002)**	-0.01 (-0.02, 0.01)	-0.01 (-0.03, 0.02)	0.49	0 (0, 0)	0.81
**Fiber**								
Richness	*Ref.*	5.41 (-2.17, 13.0)	**8.09 (0.03, 16.1)**	5.69 (-3.01, 14.4)	9.39 (-1.21, 20.0)	0.13	0.29 (-0.20, 0.79)	0.25
Shannon Index	*Ref.*	0.07 (-0.08, 0.22)	0.12 (-0.03, 0.28)	0.06 (-0.11, 0.23)	0.09 (-0.12, 0.29)	0.49	0.003 (-0.01, 0.01)	0.51
Evenness	*Ref.*	0.002 (-0.01, 0.02)	0.01 (-0.01, 0.02)	-0.001 (-0.02, 0.02)	-0.001 (-0.02, 0.02)	0.87	0 (-0.001, 0.001)	0.88

[a]Regression parameters and *P*-values are from linear regression models with specified α-diversity metric (richness, Shannon diversity index, community evenness, averaged over 100 iterations of rarefied OTU table at 3,000 sequence reads/sample) as the outcome. All models were adjusted for age, study (PLCOa, PLCOb, CPS-IIa, CPS-IIb), current smoking, BMI (mg/kg2), energy intake (kcal/day), alcohol intake (grams/day), carbohydrate intake (g/day), and GI.

[b]One star (*) indicates *P*-value < 0.05 from the linear regression model.
