# Supplementary material for: Elevated Dietary Carbohydrate and Glycemic Intake Associate with an Altered Oral Microbial Ecosystem in Two Large U.S. Cohorts
Source: Cancer Res Commun. 2022 Dec 5;2(12):1558–68. doi: 10.1158/2767-9764.CRC-22-0323 (PMC9770587; doi:10.1158/2767-9764.CRC-22-0323)
Supplement: Table S4 — Sensitivity analyses, beta diversity [file crc-22-0323-s06.pdf]

Supplementary Table S4(a). Association of β-diversity metrics with daily carbohydrates and Glycemic Index (GI) as categorical (quintiles) and continuous variables, stratified by BMI (normal: <25 kg/m^2, n=328; elevated: ≥25-<30 kg/m^2, n=358; high: ≥30 kg/m^2, n=148)

BMI Normal: <25 kg/m^2

	**Categorical**						**Continuous**
	P-value[a]						*P*-value[a]
	Q1	Q2	Q3	Q4	Q5	P-trend	Per day
Carbohydrates	*Ref.*	0.41	0.94	0.93	0.67	0.63	0.63
Glycemic Index	*Ref.*	0.66	0.56	0.30	0.45	0.43	0.38

BMI Elevated: ≥25-<30 kg/m^2

	**Categorical**						**Continuous**
	P-value[a]						*P*-value[a]
	Q1	Q2	Q3	Q4	Q5	P-trend	Per day
Carbohydrates	*Ref.*	0.90	0.90	0.92	0.88	0.93	0.57
Glycemic Index	*Ref.*	0.75	0.70	0.58	0.30	0.30	0.37

BMI High: ≥30 kg/m^2

	**Categorical**						**Continuous**
	P-value[a]						*P*-value[a]
	Q1	Q2	Q3	Q4	Q5	P-trend	Per day
Carbohydrates	*Ref.*	0.42	0.67	0.43	0.36	0.69	0.36
Glycemic Index	*Ref.*	0.62	0.40	0.48	0.57	0.37	0.72

[a]*P*-values are from PERMANOVA of weighted UniFrac distance adjusted for age, sex, study (PLCOa, PLCOb, CPS-IIa, CPS-IIb), current smoking, energy intake (kcal/day), and alcohol intake (grams/day).

Supplementary Table S4(b). Association of β-diversity metrics with daily carbohydrate and Glycemic Index (GI) as categorical (quintiles) and continuous variables, stratified by cohort (PLCO, n=441 or CPS-II, n=393)

PLCO

	**Categorical** *P*-value[a]						**Continuous** *P*-value[a]
	Q1	Q2	Q3	Q4	Q5	P-trend	Per day
Carbohydrate	*Ref.*	0.66	0.86	0.40	0.17	0.25	0.42
Glycemic Index	*Ref.*	0.12	0.78	0.19	**0.01***	**0.01***	0.11

CPS-II

	**Categorical** *P*-value[a]						**Continuous** *P*-value[a]
	Q1	Q2	Q3	Q4	Q5	P-trend	Per day
Carbohydrate	*Ref.*	0.46	0.83	0.22	0.91	0.62	0.09
Glycemic Index	*Ref.*	0.40	0.76	0.53	0.55	0.52	0.37

[a]*P*-values are from PERMANOVA of weighted UniFrac distance adjusted for age, sex, current smoking, BMI (kg/m2), energy intake (kcal/day), and alcohol intake (grams/day).

[b]One star (*) indicates *P*-value < 0.05 from the PERMANOVA model.

Supplementary Table S4(c). Association of β-diversity metrics with daily carbohydrate and Glycemic Index (GI) as categorical (quintiles) and continuous variables, stratified by sex (male, n=528 and female, n=306)

Male

	Categorical						Continuous
	P-value[a]						*P*-value[a]
	Q1	Q2	Q3	Q4	Q5	P-trend	Per day
Carbohydrates	*Ref.*	0.76	0.78	0.66	0.58	0.69	0.24
Glycemic Index	*Ref.*	0.22	0.21	0.74	0.36	0.61	0.65

Female

	Categorical						Continuous
	P-value[a]						*P*-value[a]
	Q1	Q2	Q3	Q4	Q5	P-trend	Per day
Carbohydrates	*Ref.*	0.54	0.59	0.52	0.80	0.54	0.82
Glycemic Index	*Ref.*	0.80	0.47	0.22	0.29	0.17	0.16

[a]*P*-values are from PERMANOVA of weighted UniFrac distance adjusted for age, study (PLCOa, PLCOb, CPS-IIa, CPS-IIb), current smoking, BMI (kg/m2), energy intake (kcal/day), and alcohol intake (grams/day).

Supplementary Table S4(d). Association of β-diversity metrics with daily carbohydrate and Glycemic Index (GI) as categorical (quintiles) and continuous variables in full dataset (PLCO and CPS-II cohorts) including diabetic subjects (n=938)

Full Dataset (including diabetic subjects)

	**Categorical**						**Continuous**
	P-value[a]						*P*-value[a]
	Q1	Q2	Q3	Q4	Q5	P-trend	Per day
Carbohydrates	*Ref.*	0.80	0.80	0.86	0.82	0.91	0.69
Glycemic Index	*Ref.*	0.62	0.52	0.61	0.41	0.46	0.55

[a]*P*-values are from PERMANOVA of weighted UniFrac distance adjusted for age, study (PLCOa, PLCOb, CPS-IIa, CPS-IIb), current smoking, BMI (kg/m2), energy intake (kcal/day), and alcohol intake (grams/day).

Supplementary Table S4(e). Association of β-diversity metrics with daily carbohydrate and Glycemic Index (GI) as categorical (quintiles) and continuous variables when restricting to PLCO and CPS-II cohort controls (n=543)

PLCO and CPS-II Controls

	**Categorical**						**Continuous**
	P-value[a]						*P*-value[a]
	Q1	Q2	Q3	Q4	Q5	P-trend	Per day
Carbohydrates	*Ref.*	0.2	0.25	0.34	0.75	0.79	0.6
Glycemic Index	*Ref.*	0.89	0.07	0.67	0.76	0.61	0.7

[a]*P*-values are from PERMANOVA of weighted UniFrac distance adjusted for age, study (PLCOa, PLCOb, CPS-IIa, CPS-IIb), current smoking, BMI (kg/m2), energy intake (kcal/day), and alcohol intake (grams/day).

Supplementary Table S4(f). Association of β-diversity metrics with daily carbohydrates as percent of calories as a categorical (quintiles) and continuous variable (n=834)

	**Categorical**						**Continuous**
	P-value[a]						*P*-value[a]
	Q1	Q2	Q3	Q4	Q5	P-trend	Per day
Carbohydrates as percent of calories	*Ref.*	0.90	0.26	0.55	0.60	0.58	0.39

[a]*P*-values are from PERMANOVA of weighted UniFrac distance adjusted for age, study (PLCOa, PLCOb, CPS-IIa, CPS-IIb), current smoking, BMI (kg/m2), alcohol intake (grams/day).

Supplementary Table S4(g). Association of β-diversity metrics with daily Glycemic Load (GL), Sucrose, and Fiber intake as categorical (quintiles) and continuous variables (n=834)

	**Categorical**						**Continuous**
	P-value[a]						*P*-value[a]
	Q1	Q2	Q3	Q4	Q5	P-trend	Per day
Glycemic Load	*Ref.*	0.73	0.58	0.61	0.17	0.32	0.64
Sucrose	*Ref.*	0.64	**0.02**	0.68	0.72	0.46	0.73
Fiber	*Ref.*	0.51	0.58	0.74	0.15	0.26	0.19

[a]*P*-values are from PERMANOVA of weighted UniFrac distance adjusted for age, study (PLCOa, PLCOb, CPS-IIa, CPS-IIb), current smoking, BMI (kg/m2), energy intake (kcal/day), alcohol intake (grams/day), carbohydrate intake (g/day), and GI.
