# Supplementary material for: Elevated Dietary Carbohydrate and Glycemic Intake Associate with an Altered Oral Microbial Ecosystem in Two Large U.S. Cohorts
Source: Cancer Res Commun. 2022 Dec 5;2(12):1558–68. doi: 10.1158/2767-9764.CRC-22-0323 (PMC9770587; doi:10.1158/2767-9764.CRC-22-0323)
Supplement: Table S5 — Sensitivity analyses, taxon abundance [file crc-22-0323-s07.pdf]

Supplementary Table S5(a). Association of daily carbohydrate and Glycemic Index (GI) as categorical (quintiles) or continuous variables with abundance of oral microbial taxa, stratified by BMI (normal: <25 kg/m^2, n=328; elevated: ≥25-<30 kg/m^2, n=358; high: ≥30 kg/m^2, n=148) [a]

BMI Normal: <25 kg/m^2

	Categorical								Continuous		
	Mean normalized count	Fold change[b] (95% CI)							Fold change[b] (95% CI)		
**Carbohydrates**	**Q1**		**Q2**	**Q3**	**Q4**	**Q5**	***P*-trend[c]**	**q-trend[c]**	**Per day**	***P***	**q-value**
Fusobacteria (Phylum)											
	299.81	*Ref.*	0.98 (0.76, 1.26)	1.22 (0.95, 1.58)	1.29 (0.97, 1.71)	1.39 (1.01, 1.91)	0.01	0.03	1.001 (0.999, 1.002)	0.31	0.39
Actinobacteria (Phylum)											
	1479.01	*Ref.*	1 (0.79, 1.25)	0.85 (0.68, 1.08)	0.85 (0.66, 1.11)	0.68 (0.51, 0.91)	0.01	0.03	0.999 (0.998, 1)	0.05	0.17
Fusobacteria; Fusobacteriia (Class)											
	294.22	*Ref.*	1.11 (0.87, 1.43)	1.27 (0.99, 1.63)	1.36 (1.03, 1.8)	1.64 (1.2, 2.24)	0.001	0.02	1.001 (1, 1.003)	0.02	0.18
Actinobacteria; Coriobacteriia (Class)											
	52.36	*Ref.*	0.91 (0.63, 1.32)	0.82 (0.56, 1.19)	0.72 (0.48, 1.09)	0.56 (0.35, 0.9)	0.01	0.09	0.997 (0.995, 0.999)	4.4E-4	0.01
Fusobacteria; Fusobacteriia; Fusobacteriales (Order)											
	307.54	*Ref.*	1.06 (0.81, 1.38)	1.35 (1.04, 1.76)	1.39 (1.03, 1.87)	1.68 (1.2, 2.34)	4.9E-4	0.01	1.002 (1, 1.003)	0.03	0.26
Actinobacteria; Coriobacteriia; Coriobacteriales (Order)											
	55.35	*Ref.*	0.84 (0.57, 1.24)	0.86 (0.59, 1.27)	0.68 (0.44, 1.05)	0.56 (0.35, 0.91)	0.02	0.19	0.997 (0.995, 0.999)	0.001	0.03
Actinobacteria; Coriobacteriia; Coriobacteriales; *Coriobacteriaceae* (Family)											
	51.86	*Ref.*	0.89 (0.6, 1.31)	0.87 (0.58, 1.28)	0.61 (0.4, 0.95)	0.56 (0.34, 0.91)	0.01	0.17	0.996 (0.994, 0.998)	3.0E-4	0.01
Firmicutes; Bacilli; Lactobacillales; *Streptococcaceae; Streptococcus; sp._oral_taxon_423* (Secies)											
	1.87	*Ref.*	1.06 (0.65, 1.74)	1.49 (0.92, 2.42)	2.05 (1.21, 3.48)	2.2 (1.21, 4.02)	0.001	0.06	1.004 (1.002, 1.007)	0.001	0.04
Firmicutes; Bacilli; Lactobacillales; *Streptococcaceae; Streptococcus; mitis* (Species)											
	8.47	*Ref.*	1.4 (0.97, 2.02)	1.47 (1.02, 2.11)	1.78 (1.19, 2.67)	2.1 (1.33, 3.31)	0.001	0.06	1.003 (1.001, 1.005)	4.0E-4	0.02
Actinobacteria; Coriobacteriia; Coriobacteriales; *Coriobacteriaceae; Atopobium; parvulum* (Species)											
	39.72	*Ref.*	0.85 (0.57, 1.26)	0.81 (0.55, 1.21)	0.45 (0.29, 0.71)	0.5 (0.3, 0.82)	3.9E-4	0.06	0.996 (0.994, 0.998)	9.0E-05	0.01
Actinobacteria; Actinobacteria; Actinomycetales; *Actinomycetaceae; Actinomyces; sp._oral_taxon_180* (OTU)											
	20.42	*Ref.*	1.2 (0.63, 2.27)	1.32 (0.69, 2.49)	0.41 (0.2, 0.83)	0.39 (0.17, 0.87)	5.0E-04	0.05	0.995 (0.992, 0.998)	0.003	0.11
Fusobacteria; Fusobacteriia; Fusobacteriales; *Fusobacteriaceae; Fusobacterium; nucleatum_subsp._vincentii* (OTU)											
	20.26	*Ref.*	2.09 (1.26, 3.48)	2.47 (1.49, 4.1)	2.08 (1.18, 3.67)	3.63 (1.92, 6.83)	0.001	0.05	1.003 (1.001, 1.006)	0.01	0.24

Firmicutes; Bacilli; Lactobacillales; *Streptococcaceae; Streptococcus; sp._oral_taxon_423* (OTU)										
1.85	*Ref.*	1.07 (0.65, 1.75)	1.5 (0.92, 2.44)	2.07 (1.22, 3.52)	2.18 (1.2, 3.99)	0.001	0.05	1.004 (1.002, 1.007)	0.001	0.03
Firmicutes; Bacilli; Lactobacillales; *Streptococcaceae; Streptococcus; mitis* (OTU)										
8.44	*Ref.*	1.41 (0.98, 2.03)	1.47 (1.02, 2.11)	1.8 (1.2, 2.7)	2.13 (1.35, 3.35)	0.001	0.05	1.003 (1.002, 1.005)	3.0E-04	0.02
Actinobacteria; Coriobacteriia; Coriobacteriales; *Coriobacteriaceae; Atopobium; parvulum* (OTU)										
39.23	*Ref.*	0.88 (0.6, 1.31)	0.84 (0.57, 1.25)	0.46 (0.3, 0.72)	0.5 (0.3, 0.81)	3.4E-4	0.05	0.996 (0.994, 0.998)	1.0E-04	0.02

**Glycemic Index**	**Q1**	**Q2**	**Q3**	**Q4**	**Q5**	***P*-trend[c]**	**q-trend[c]**	**Per day**	***P***	**q-value**
Actinobacteria; Coriobacteriia (Class)										
52.36	*Ref.*	0.81 (0.57, 1.16)	0.87 (0.62, 1.23)	0.68 (0.47, 0.98)	0.48 (0.33, 0.69)	1.3E-4	0.002	0.94 (0.9, 0.97)	0.001	0.01
Actinobacteria; Coriobacteriia; Eggerthellales (Order)										
0.63	*Ref.*	0.47 (0.12, 1.8)	0.25 (0.07, 0.91)	0.23 (0.06, 0.91)	0.31 (0.08, 1.27)	NA[d]	NA[d]	0.82 (0.72, 0.94)	0.004	0.04
Firmicutes; Bacilli; Bacillales (Order)										
310.39	*Ref.*	1.29 (0.94, 1.77)	1.26 (0.93, 1.72)	1.82 (1.32, 2.52)	1.67 (1.2, 2.31)	1.4E-4	0.003	1.05 (1.02, 1.09)	0.002	0.03
Firmicutes; Bacilli; Lactobacillales (Order)										
5877.12	*Ref.*	1.22 (0.97, 1.52)	1.17 (0.94, 1.45)	1.23 (0.98, 1.55)	1.41 (1.12, 1.77)	0.01	0.04	1.03 (1.01, 1.05)	0.01	0.07
Actinobacteria; Coriobacteriia; Coriobacteriales (Order)										
55.35	*Ref.*	0.81 (0.56, 1.17)	1.01 (0.7, 1.44)	0.76 (0.52, 1.11)	0.52 (0.35, 0.76)	0.003	0.03	0.95 (0.91, 0.99)	0.01	0.05
Firmicutes; Bacilli; Bacillales; *Gemellaceae* (Family)										
308.26	*Ref.*	1.28 (0.93, 1.76)	1.21 (0.89, 1.65)	1.78 (1.29, 2.47)	1.69 (1.22, 2.36)	1.5E-4	0.01	1.06 (1.02, 1.09)	0.001	0.04
Firmicutes; Bacilli; Bacillales; *Gemellaceae; Gemella* (Genus)										
321.36	*Ref.*	1.25 (0.89, 1.75)	1.18 (0.85, 1.63)	1.76 (1.25, 2.47)	1.66 (1.18, 2.35)	3.5E-4	0.03	1.06 (1.02, 1.09)	0.002	0.15
Actinobacteria; Actinobacteria; Actinomycetales; *Actinomycetaceae; Actinomyces; lingnae_[NVP]* (Species)										
9.71	*Ref.*	0.82 (0.53, 1.27)	0.77 (0.5, 1.17)	0.67 (0.43, 1.04)	0.43 (0.27, 0.68)	3.1E-4	0.04	0.92 (0.88, 0.97)	5.3E-4	0.15
Firmicutes; Negativicutes; Selenomonadales; *Veillonellaceae; Veillonella; denticariosi* (Species)										
3.48	*Ref.*	1.08 (0.56, 2.07)	1.15 (0.61, 2.16)	4.31 (2.27, 8.2)	3 (1.55, 5.8)	8.8E-07	2.5E-4	1.15 (1.07, 1.23)	NA[d]	NA[d]

BMI Elevated: ≥25-<30 kg/m^2

	Categorical		Continuous
**Mean normalized count**	**Fold change[b] (95% CI)**		**Fold change[b] (95% CI)**

Carbohydrates	Q1	Q2	Q3	Q4	Q5	*P*-trend[c]	q-trend[c]	Per day	*P*	q-value
Fusobacteria; Fusobacteriia; Fusobacteriales; *Leptotrichiaceae; Leptotrichia; hongkongensis* (Species)										
11.3	*Ref.*	0.74 (0.42, 1.29)	1.15 (0.63, 2.11)	1.06 (0.58, 1.92)	3.29 (1.67, 6.49)	0.01	0.53	1.006 (1.003, 1.008)	1.6E-4	0.04
Fusobacteria; Fusobacteriia; Fusobacteriales; *Leptotrichiaceae; Leptotrichia; hongkongensis* (OTU)										
11.36	*Ref.*	0.76 (0.44, 1.34)	1.21 (0.66, 2.22)	1.08 (0.6, 1.96)	3.43 (1.74, 6.77)	0.004	0.55	1.006 (1.003, 1.009)	1.2E-4	0.04
**Glycemic Index**	**Q1**	**Q2**	**Q3**	**Q4**	**Q5**	***P*-trend[c]**	**q-trend[c]**	**Per day**		
No taxa overlapping with the full analysis										
-	*Ref.*	-	-	-	-	-	-	-	-	-

BMI High: ≥30 kg/m^2

	Categorical							Continuous		
Mean normalized count		Fold change[b] (95% CI)						Fold change[b] (95% CI)		
**Carbohydrate**	**Q1**	**Q2**	**Q3**	**Q4**	**Q5**	***P*-trend[c]**	**q-trend[c]**	**Per day**	***P***	**q-value**
Actinobacteria; Coriobacteriia; Coriobacteriales; *Atopobiaceae* (Family)										
1.42	*Ref.*	1.36 (0.56, 3.28)	0.6 (0.24, 1.51)	0.56 (0.21, 1.5)	0.23 (0.07, 0.78)	0.01	0.1	0.993 (0.988, 0.997)	0.002	0.02
Actinobacteria; Coriobacteriia; Coriobacteriales; *Atopobiaceae; Olsenella* (Genus)										
1.46	*Ref.*	1.33 (0.55, 3.21)	0.56 (0.22, 1.4)	0.55 (0.21, 1.46)	0.23 (0.07, 0.76)	0.01	0.12	0.993 (0.988, 0.997)	0.001	0.02
**Glycemic Index**	**Q1**	**Q2**	**Q3**	**Q4**	**Q5**			**Per day**		
-[e]										
-	*Ref.*	-	-	-	-	-	-	-	-	-

[a]Taxa included in the table were associated with daily carbohydrate or GI at q-trend <0.05 for categorical variables or q-value < 0.05 for continuous variables and were significant (q<0.05) in the full cohort analysis (n=834) at or below the family level

[b]DESeq2 models adjusted for age, sex, study (PLCOa, PLCOb, CPS-IIa, CPS-IIb), current smoking, BMI (kg/m2), energy intake (kcal/day), and alcohol intake (grams/day).

[c]Trend tests across quintiles were calculated by entering the categorical variables into the models as continuous terms

[d]*P* not calculated due to heavy outlier influence on model (maximum Cook's distance > 10).

[e]No taxa met the q<0.05 threshold for significance

Supplementary Table S5(b). Association of daily carbohydrate and Glycemic Index (GI) as categorical (quintiles) or continuous variables with abundance of oral microbial taxa, stratified by cohort (PLCO, n=441 or CPS-II, n=393) [a]

PLCO

		Categorical						Continuous		
**Mean normalized count**		**Fold change[b] (95% CI)**						**Fold change[b] (95% CI)**		
**Carbohydrates**	**Q1**	**Q2**	**Q3**	**Q4**	**Q5**	***P*-trend[c]**	**q-trend[c]**	**Per day**	***P***	**q-value**
Firmicutes (Phylum)										
9196.72	*Ref.*	1.11 (0.9, 1.36)	0.89 (0.73, 1.1)	0.89 (0.73, 1.12)	1.21 (0.96, 1.52)	7.1E-05	5.0E-04	1.04 (1.01, 1.06)	NA[d]	NA[d]
Firmicutes; Bacilli (Class)										
8600.02	*Ref.*	1 (0.79, 1.26)	0.9 (0.71, 1.13)	0.9 (0.61, 0.99)	1.21 (0.93, 1.57)	4.7E-06	7.5E-05	1.04 (1.02, 1.07)	0.001	0.02
Actinobacteria; Coriobacteriia (Class)										
45.22	*Ref.*	1.06 (0.78, 1.43)	0.73 (0.54, 0.98)	0.73 (0.67, 1.25)	0.69 (0.49, 0.97)	0.003	0.02	0.96 (0.93, 0.99)	0.01	0.09
Firmicutes; Bacilli; Bacillales (Order)										
404.03	*Ref.*	0.76 (0.56, 1.04)	0.92 (0.68, 1.25)	0.92 (0.5, 0.95)	1.05 (0.74, 1.48)	7.4E-4	0.01	1.05 (1.02, 1.08)	0.002	0.02
Firmicutes; Bacilli; Lactobacillales (Order)										
7521.96	*Ref.*	1.2 (0.97, 1.49)	1.03 (0.83, 1.27)	1.03 (0.7, 1.09)	1.2 (0.94, 1.53)	2.0E-06	4.3E-05	1.04 (1.02, 1.07)	9.1E-05	0.002
Actinobacteria; Coriobacteriia; Coriobacteriales (Order)										
46.72	*Ref.*	1.04 (0.77, 1.42)	0.75 (0.55, 1.02)	0.75 (0.65, 1.24)	0.63 (0.45, 0.9)	6.5E-4	0.01	0.96 (0.93, 0.99)	0.01	0.03
Firmicutes; Bacilli; Bacillales; *Gemellaceae* (Family)										
403.6	*Ref.*	0.74 (0.54, 1.01)	0.84 (0.62, 1.15)	0.84 (0.48, 0.91)	1.03 (0.72, 1.46)	2.3E-4	0.004	1.05 (1.02, 1.08)	0.002	0.03
Firmicutes; Bacilli; Lactobacillales; *Streptococcaceae* (Family)										
6638.7	*Ref.*	1.06 (0.87, 1.3)	1.05 (0.86, 1.28)	1.05 (0.77, 1.17)	1.27 (1.02, 1.59)	6.5E-06	2.2E-4	1.04 (1.01, 1.06)	7.0E-04	0.02
Actinobacteria; Coriobacteriia; Coriobacteriales; *Coriobacteriaceae* (Family)										
45.9	*Ref.*	1.07 (0.78, 1.47)	0.71 (0.52, 0.98)	0.71 (0.64, 1.25)	0.58 (0.4, 0.83)	0.001	0.02	0.95 (0.92, 0.99)	0.01	0.04
Firmicutes; Bacilli; Bacillales; *Gemellaceae; Gemella* (Genus)										
410.38	*Ref.*	0.78 (0.57, 1.07)	0.83 (0.6, 1.14)	0.83 (0.48, 0.93)	1.03 (0.72, 1.47)	0.001	0.03	1.04 (1.01, 1.08)	0.01	0.13
Firmicutes; Bacilli; Lactobacillales; *Streptococcaceae; Streptococcus* (Genus)										
6627.77	*Ref.*	1.07 (0.87, 1.3)	1.04 (0.85, 1.27)	1.04 (0.77, 1.18)	1.28 (1.03, 1.61)	9.7E-05	0.01	1.03 (1.01, 1.05)	0.004	0.1
Actinobacteria; Coriobacteriia; Coriobacteriales; *Coriobacteriaceae; Atopobium* (Genus)										

44.18	*Ref.*	1.13 (0.83, 1.55)	0.77 (0.56, 1.05)	0.77 (0.69, 1.32)	0.63 (0.44, 0.9)	0.001	0.03	0.95 (0.92, 0.99)	0.01	0.1
Actinobacteria; Actinobacteria; Actinomycetales; *Actinomycetaceae; Actinomyces; sp._oral_taxon_180* (Species)										
133.19	*Ref.*	0.89 (0.67, 1.17)	0.9 (0.68, 1.19)	0.9 (0.82, 1.47)	0.71 (0.52, 0.97)	0.00064	0.05	0.96 (0.94, 0.99)	0.01	0.28
Firmicutes; Bacilli; Lactobacillales; *Streptococcaceae; Streptococcus; salivarius* (Species)										
1198.25	*Ref.*	1.25 (0.91, 1.72)	1.23 (0.89, 1.68)	1.23 (0.86, 1.65)	1.58 (1.11, 2.26)	0.00038	0.05	1.04 (1.01, 1.08)	0.01	0.28
Actinobacteria; Actinobacteria; Actinomycetales; *Actinomycetaceae; Actinomyces; sp._oral_taxon_180* (OTU)										
23.69	*Ref.*	0.68 (0.39, 1.17)	1.01 (0.59, 1.75)	1.01 (0.46, 1.43)	0.45 (0.25, 0.84)	4.9E-05	0.01	0.91 (0.86, 0.96)	7.5E-4	0.17
Actinobacteria; Actinobacteria; Actinomycetales; *Actinomycetaceae; Actinomyces; lingnae_[NVP]* (OTU)										
11.11	*Ref.*	1.5 (1.03, 2.2)	0.98 (0.67, 1.44)	0.98 (0.96, 2.13)	1.12 (0.73, 1.72)	0.00058	0.05	0.95 (0.91, 0.98)	0.004	0.22
Firmicutes; Bacilli; Lactobacillales; *Streptococcaceae; Streptococcus; salivarius* (OTU)										
1214.47	*Ref.*	1.28 (0.94, 1.76)	1.24 (0.9, 1.7)	1.24 (0.81, 1.57)	1.57 (1.1, 2.24)	0.00044	0.05	1.04 (1.01, 1.08)	0.01	0.23

**Glycemic Index**	**Q1**	**Q2**	**Q3**	**Q4**	**Q5**	***P*-trend**[c]	**q-trend**[c]	**Per day**	***P***	**q-value**
Fusobacteria; Fusobacteriia; Fusobacteriales; *Leptotrichiaceae* (Family)										
112.2	*Ref.*	0.95 (0.7, 1.29)	0.92 (0.68, 1.25)	0.98 (0.72, 1.33)	1.02 (0.75, 1.4)	0.0018	0.05	1 (1, 1)	0.01	0.11
Actinobacteria; Coriobacteriia; Coriobacteriales; *Coriobacteriaceae* (Family)										
45.94	*Ref.*	1.04 (0.75, 1.43)	0.77 (0.56, 1.07)	0.97 (0.7, 1.34)	0.58 (0.42, 0.81)	0.0028	0.05	1 (1, 1)	0.01	0.11

CPS-II

	**Categorical**							**Continuous**		
**Mean normalized count**		**Fold change**[b] **(95% CI)**						**Fold change**[b] **(95% CI)**		
**Carbohydrate**	**Q1**	**Q2**	**Q3**	**Q4**	**Q5**	***P*-trend**[c]	**q-trend**[c]	**Per day**	***P***	**q-value**
Actinobacteria; Actinobacteria; Actinomycetales; *Actinomycetaceae; Actinomyces; viscosus* (Species)										
1.85	*Ref.*	2.01 (1.08, 3.76)	1.79 (0.87, 3.68)	1.65 (0.73, 3.72)	2.03 (0.65, 6.35)	0.002	0.2	0.91 (0.86, 0.97)	0.003	0.36
Firmicutes; Negativicutes; Selenomonadales; *Veillonellaceae; Veillonella; denticariosi* (Species)										
5.12	*Ref.*	0.79 (0.46, 1.36)	0.96 (0.52, 1.79)	0.68 (0.34, 1.37)	1.09 (0.41, 2.93)	0.004	0.2	1.08 (1.03, 1.14)	0.004	0.36
Firmicutes; Negativicutes; Selenomonadales; *Veillonellaceae; Selenomonas; noxia* (Species)										
2.7	*Ref.*	1.23 (0.72, 2.13)	1.25 (0.67, 2.34)	1 (0.49, 2.03)	0.77 (0.28, 2.09)	0.004	0.2	0.93 (0.88, 0.98)	0.01	0.44
Actinobacteria; Actinobacteria; Actinomycetales; *Actinomycetaceae; Actinomyces; viscosus* (OTU)										
1.89	*Ref.*	1.98 (1.06, 3.72)	1.77 (0.85, 3.68)	1.63 (0.72, 3.7)	2.1 (0.67, 6.62)	0.001	0.12	0.91 (0.86, 0.97)	0.002	0.3
Firmicutes; Negativicutes; Selenomonadales; *Veillonellaceae; Selenomonas; noxia* (OTU)										

1.14	*Ref.*	1.67 (0.74, 3.75)	2.29 (0.91, 5.79)	1.66 (0.58, 4.75)	0.78 (0.18, 3.47)	0.004	0.21	0.9 (0.83, 0.97)	0.01	0.33
**Glycemic Index**	**Q1**	**Q2**	**Q3**	**Q4**	**Q5**	***P*-trend[c]**	**q-trend[c]**	**Per day**	***P***	**q-value**
Actinobacteria; Actinobacteria; Actinomycetales; *Actinomycetaceae; Actinomyces; sp._oral_taxon_169* (Species)										
46.61	*Ref.*	1.45 (0.87, 2.4)	1.44 (0.87, 2.39)	1.13 (0.68, 1.88)	1.25 (0.74, 2.1)	0.01	0.28	1.01 (1, 1.01)	9.7E-05	0.02
Actinobacteria; Actinobacteria; Actinomycetales; *Actinomycetaceae; Actinomyces; graevenitzii* (Species)										
45.01	*Ref.*	1.2 (0.74, 1.96)	1.08 (0.67, 1.76)	0.86 (0.52, 1.4)	1.16 (0.7, 1.91)	0.003	0.17	0.99 (0.99, 1)	3.8E-4	0.03
Actinobacteria; Actinobacteria; Actinomycetales; *Actinomycetaceae; Actinomyces; sp._oral_taxon_169* (OTU)										
46.66	*Ref.*	1.5 (0.91, 2.49)	1.47 (0.89, 2.43)	1.11 (0.67, 1.84)	1.22 (0.73, 2.05)	0.01	0.3	1.01 (1, 1.01)	1.5E-4	0.02
Actinobacteria; Actinobacteria; Actinomycetales; *Actinomycetaceae; Actinomyces; sp._oral_taxon_180* (OTU)										
19.61	*Ref.*	0.88 (0.51, 1.52)	1.75 (1.02, 3.02)	0.93 (0.53, 1.61)	1.23 (0.7, 2.16)	3.4E-05	0.01	0.99 (0.99, 1)	1.7E-4	0.09
Actinobacteria; Actinobacteria; Actinomycetales; *Actinomycetaceae; Actinomyces; graevenitzii* (OTU)										
44.92	*Ref.*	1.16 (0.71, 1.88)	1.06 (0.65, 1.72)	0.84 (0.52, 1.38)	1.12 (0.68, 1.85)	0.002	0.12	0.99 (0.99, 1)	2.8E-4	0.02

[a]Taxa included in the table were associated with daily carbohydrate or GI at q-trend <0.05 for categorical variables or q-value < 0.05 for continuous variables and were significant (q<0.05) in the full cohort analysis (n=834) at or below the family level

[b]DESeq2 models adjusted for age, sex, study (PLCOa, PLCOb, CPS-IIa, CPS-IIb,), current smoking, BMI (kg/m2), energy intake (kcal/day), and alcohol intake (grams/day).

[c]Trend tests across quintiles were calculated by entering the categorical variables into the models as continuous terms

[d]*P* not calculated due to heavy outlier influence on model (maximum Cook's distance > 10).

Supplementary Table S5(c). Association of daily carbohydrates and Glycemic Index (GI) as categorical (quintiles) or continuous variables with abundance of oral microbial taxa, stratified by sex (male, n=528 and female, n=306) [a]

Male

	**Categorical**								**Continuous**		
	**Mean normalized count**		**Fold change[b] (95% CI)**						**Fold change[b] (95% CI)**		
**Carbohydrates**		**Q1**	**Q2**	**Q3**	**Q4**	**Q5**	***P*-trend[c]**	**q-trend[c]**	**Per day**	***P***	**q-value**
Fusobacteria (Phylum)											
	266.7	*Ref.*	1.15 (0.93, 1.43)	1.32 (1.07, 1.62)	1.39 (1.12, 1.72)	1.45 (1.14, 1.85)	0.001	0.01	1.001 (1, 1.002)	0.12	0.37
Fusobacteria; Fusobacteriia (Class)											
	260.68	*Ref.*	1.18 (0.95, 1.46)	1.36 (1.11, 1.67)	1.36 (1.1, 1.67)	1.52 (1.2, 1.93)	5.5E-4	0.01	1.001 (1, 1.002)	0.02	0.11
Actinobacteria; Coriobacteriia; Coriobacteriales (Order)											
	54.22	*Ref.*	1.03 (0.75, 1.41)	0.81 (0.6, 1.1)	0.91 (0.67, 1.25)	0.55 (0.38, 0.78)	0.001	0.03	0.998 (0.997, 0.999)	0.003	0.07
Actinobacteria; Coriobacteriia; Coriobacteriales; *Coriobacteriaceae* (Family)											
	51.79	*Ref.*	1.11 (0.8, 1.53)	0.82 (0.6, 1.12)	0.9 (0.65, 1.24)	0.54 (0.38, 0.78)	7.1E-4	0.03	0.998 (0.996, 0.999)	0.002	0.06
Actinobacteria; Coriobacteriia; Coriobacteriales; *Coriobacteriaceae; Atopobium* (Genus)											
	48.95	*Ref.*	1.12 (0.81, 1.53)	0.84 (0.62, 1.14)	0.86 (0.63, 1.18)	0.57 (0.4, 0.82)	5.1E-4	0.04	0.998 (0.996, 0.999)	0.002	0.09
Fusobacteria; Fusobacteriia; Fusobacteriales; *Leptotrichiaceae; Leptotrichia; hongkongensis* (Species)											
	11.63	*Ref.*	0.93 (0.57, 1.54)	1.2 (0.74, 1.94)	1.65 (1.01, 2.7)	2.26 (1.3, 3.93)	4.3E-4	0.09	1.004 (1.002, 1.006)	1.2E-4	0.02
Firmicutes; Negativicutes; Selenomonadales; *Veillonellaceae; Veillonella; denticariosi* (Species)											
	2.95	*Ref.*	1.34 (0.76, 2.34)	1.7 (0.99, 2.91)	1.83 (1.05, 3.17)	2.43 (1.31, 4.54)	0.004	0.15	1.005 (1.002, 1.007)	1.7E-4	0.02
Actinobacteria; Actinobacteria; Actinomycetales; *Actinomycetaceae; Actinomyces; sp._oral_taxon_180* (OTU)											
	24.96	*Ref.*	1 (0.59, 1.71)	0.67 (0.4, 1.13)	0.49 (0.29, 0.83)	0.35 (0.19, 0.63)	4.0E-05	0.01	0.995 (0.993, 0.997)	3.9E-05	0.01
Fusobacteria; Fusobacteriia; Fusobacteriales; *Leptotrichiaceae; Leptotrichia; hongkongensis* (OTU)											
	11.71	*Ref.*	0.93 (0.57, 1.54)	1.23 (0.76, 1.99)	1.69 (1.04, 2.77)	2.25 (1.29, 3.91)	4.0E-04	0.06	1.004 (1.002, 1.006)	1.0E-04	0.01
Firmicutes; Negativicutes; Selenomonadales; *Veillonellaceae; Veillonella; denticariosi* (OTU)											
	2.92	*Ref.*	1.33 (0.76, 2.33)	1.68 (0.98, 2.87)	1.79 (1.03, 3.1)	2.45 (1.31, 4.56)	0.004	0.17	1.005 (1.002, 1.007)	1.5E-4	0.01
**Glycemic Index**		**Q1**	**Q2**	**Q3**	**Q4**	**Q5**			**Per day**		
Actinobacteria; Coriobacteriia (Class)											
	51.87	*Ref.*	1.12 (0.85, 1.49)	0.83 (0.63, 1.1)	0.8 (0.6, 1.06)	0.71 (0.53, 0.95)	0.001	0.02	0.98 (0.95, 1.01)	0.16	0.46
Actinobacteria; Coriobacteriia; Coriobacteriales (Order)											
	54.22	*Ref.*	1.1 (0.82, 1.49)	0.84 (0.62, 1.12)	0.83 (0.61, 1.12)	0.71 (0.53, 0.96)	0.003	0.04	0.98 (0.95, 1.01)	0.12	0.45

Firmicutes; Negativicutes; Selenomonadales; *Veillonellaceae; Veillonella; denticariosi* (Species)											
	2.95	*Ref.*	1.98 (1.2, 3.29)	1.64 (0.99, 2.73)	1.47 (0.88, 2.47)	2.69 (1.62, 4.46)	0.003	0.14	1.09 (1.04, 1.15)	1.9E-4	0.05
Firmicutes; Negativicutes; Selenomonadales; *Veillonellaceae; Veillonella; denticariosi* (OTU)											
	2.92	*Ref.*	1.97 (1.19, 3.27)	1.66 (1, 2.76)	1.45 (0.86, 2.43)	2.7 (1.63, 4.48)	0.003	0.16	1.1 (1.04, 1.15)	1.7E-4	0.05

Female

	Mean normalized count	Categorical: Fold change[b] (95% CI)							Continuous: Fold change[b] (95% CI)		
							P-trend[c]	q-trend[c]		*P*	q-value
**Carbohydrates**		**Q1**	**Q2**	**Q3**	**Q4**	**Q5**			**Per day**		
Firmicutes; Bacilli; Bacillales (Order)											
	417.41	*Ref.*	0.87 (0.64, 1.19)	0.54 (0.38, 0.77)	0.62 (0.41, 0.94)	0.5 (0.3, 0.83)	0.001	0.03	0.996 (0.994, 0.998)	4.4E-4	0.01
Firmicutes; Bacilli; Bacillales; *Gemellaceae* (Family)											
	420.73	*Ref.*	0.84 (0.61, 1.15)	0.55 (0.39, 0.8)	0.6 (0.4, 0.91)	0.52 (0.31, 0.86)	0.002	0.06	0.996 (0.994, 0.998)	8.2E-4	0.03
**Glycemic Index**		**Q1**	**Q2**	**Q3**	**Q4**	**Q5**	***P*-trend[c]**	**q-trend[c]**	**Per day**		
Firmicutes (Phylum)											
	7409.32	*Ref.*	1.02 (0.84, 1.24)	1.2 (0.99, 1.46)	1.41 (1.17, 1.69)	1.29 (1.06, 1.58)	9.3E-05	6.5E-4	1.03 (1.01, 1.06)	9.9E-4	0.01
Firmicutes; Bacilli (Class)											
	6814.62	*Ref.*	1.02 (0.81, 1.29)	1.26 (1, 1.59)	1.4 (1.12, 1.75)	1.32 (1.03, 1.67)	7.7E-4	0.01	1.03 (1.01, 1.06)	0.01	0.11
Firmicutes; Bacilli; Bacillales (Order)											
	417.41	*Ref.*	1.06 (0.76, 1.5)	1.01 (0.72, 1.41)	2.09 (1.51, 2.9)	1.5 (1.05, 2.14)	6.9E-05	0.001	1.06 (1.02, 1.1)	0.001	0.03
Firmicutes; Bacilli; Lactobacillales (Order)											
	6262.83	*Ref.*	1.04 (0.82, 1.3)	1.29 (1.03, 1.61)	1.33 (1.07, 1.66)	1.25 (0.99, 1.58)	0.01	0.05	1.03 (1.01, 1.06)	0.01	0.11
Firmicutes; Bacilli; Bacillales; *Gemellaceae* (Family)											
	420.73	*Ref.*	1.02 (0.72, 1.44)	0.93 (0.66, 1.3)	2.08 (1.5, 2.88)	1.54 (1.08, 2.19)	4.9E-05	0.002	1.06 (1.03, 1.1)	6.8E-4	0.02
Firmicutes; Bacilli; Bacillales; *Gemellaceae; Gemella* (Genus)											
	427.53	*Ref.*	0.97 (0.68, 1.39)	0.94 (0.66, 1.33)	1.98 (1.41, 2.76)	1.52 (1.06, 2.19)	1.1E-4	0.01	1.06 (1.02, 1.1)	0.002	0.13
Firmicutes; Bacilli; Bacillales; *Gemellaceae; Gemella; haemolysans* (Species)											
	320.59	*Ref.*	1.26 (0.83, 1.92)	1.02 (0.67, 1.55)	2.3 (1.54, 3.43)	1.95 (1.26, 3.02)	3.6E-05	0.01	1.08 (1.03, 1.13)	7.8E-4	0.1
Firmicutes; Bacilli; Bacillales; *Gemellaceae; Gemella; haemolysans* (OTU)											
	316.73	*Ref.*	1.29 (0.84, 1.96)	1.01 (0.67, 1.53)	2.26 (1.51, 3.37)	1.98 (1.28, 3.06)	4.1E-05	0.01	1.08 (1.03, 1.13)	8.2E-4	0.12

[a]Taxa included in the table were associated with daily GI or GL at q-trend <0.05 for categorical variables or q-value < 0.05 for continuous variables and were significant (q<0.05) in the full cohort analysis (n=834) at or below the family level

[b]DESeq2 models adjusted for age, sex, study (PLCOa, PLCOb, CPS-IIa, CPS-IIb), current smoking, BMI (kg/m2), energy intake (kcal/day), and alcohol intake (grams/day)

[c]Trend tests across quintiles were calculated by entering the categorical variables into the models as continuous terms

Supplementary Table S5(d). Association of daily carbohydrate and Glycemic Index (GI) as categorical (quintiles) or continuous variables with abundance of oral microbial taxa in full dataset (PLCO and CPS-II cohorts) including diabetic subjects (n=938)[a]

Full Dataset (including diabetic subjects)

Mean normalized count	Categorical: Fold change[b] (95% CI)							Continuous: Fold change[b] (95% CI)		
**Carbohydrates**	**Q1**	**Q2**	**Q3**	**Q4**	**Q5**	***P*-trend[c]**	**q-trend[c]**	**Per day**	***P***	**q-value**
Fusobacteria; Fusobacteriia; Fusobacteriales; Leptotrichiaceae (Family)										
123.22	*Ref.*	0.89 (0.73, 1.08)	1.06 (0.88, 1.29)	0.92 (0.76, 1.13)	1.21 (0.99, 1.47)	0.0015	0.057	1.17 (1.07, 1.27)	2.8E-04	0.01
Fusobacteria; Fusobacteriia; Fusobacteriales; Leptotrichiaceae; Leptotrichia (Genus)										
122.65	*Ref.*	0.88 (0.72, 1.07)	1.01 (0.83, 1.23)	0.91 (0.75, 1.11)	1.18 (0.97, 1.43)	0.002	0.15	1.16 (1.07, 1.26)	5.2E-04	0.038
Firmicutes; Bacilli; Lactobacillales; Streptococcaceae; Streptococcus; sp._oral_taxon_056 (Species)										
59.75	*Ref.*	1.02 (0.83, 1.26)	0.92 (0.75, 1.13)	1.07 (0.86, 1.32)	1.14 (0.92, 1.41)	0.0021	0.18	1.19 (1.09, 1.3)	1.6E-04	0.04
Firmicutes; Bacilli; Lactobacillales; Streptococcaceae; Streptococcus; sp._oral_taxon_056 (OTU)										
59.02	*Ref.*	1.01 (0.82, 1.24)	0.92 (0.75, 1.13)	1.06 (0.86, 1.31)	1.16 (0.94, 1.43)	0.0017	0.13	1.19 (1.09, 1.3)	7.0E-05	0.011
Actinobacteria; Actinobacteria; Actinomycetales; Actinomycetaceae; Actinomyces; sp._oral_taxon_180 (OTU)										
22.75	*Ref.*	1.04 (0.72, 1.51)	1.18 (0.82, 1.69)	1.04 (0.72, 1.52)	0.92 (0.63, 1.34)	4.70E-05	0.015	0.7 (0.6, 0.82)	6.8E-06	0.0021
**Glycemic Index**	**Q1**	**Q2**	**Q3**	**Q4**	**Q5**	***P*-trend[c]**	**q-trend[c]**	**Per day**	***P***	**q-value**
Actinobacteria; Coriobacteriia (Class)										
50.65	*Ref.*	1.04 (0.85, 1.28)	0.89 (0.72, 1.11)	0.94 (0.75, 1.18)	0.79 (0.61, 1.02)	5.3E-05	9.1E-04	0.92 (0.86, 0.99)	0.023	0.19
Actinobacteria; Coriobacteriia; Eggerthellales (Order)										
0.47	*Ref.*	1.01 (0.5, 2.03)	0.57 (0.27, 1.19)	0.84 (0.39, 1.81)	0.62 (0.26, 1.49)	0.0036	0.025	1.04 (1.02, 1.07)	0.001	0.02
Firmicutes; Bacilli; Bacillales (Order)										
329.14	*Ref.*	0.77 (0.64, 0.93)	0.82 (0.67, 1)	0.72 (0.58, 0.88)	0.79 (0.62, 1.01)	8.6E-04	0.0091	1.09 (1.02, 1.16)	0.0071	0.074
Actinobacteria; Coriobacteriia; Coriobacteriales (Order)										
52.26	*Ref.*	1.05 (0.85, 1.29)	0.92 (0.73, 1.14)	0.93 (0.74, 1.17)	0.72 (0.55, 0.94)	3.00E-04	0.0062	0.93 (0.86, 0.99)	0.032	0.22
Firmicutes; Bacilli; Bacillales; Gemellaceae (Family)										
331.69	*Ref.*	0.75 (0.62, 0.91)	0.78 (0.63, 0.95)	0.69 (0.56, 0.86)	0.79 (0.62, 1.01)	0.0015	0.028	1.09 (1.02, 1.17)	0.0077	0.23
Actinobacteria; Coriobacteriia; Coriobacteriales; Coriobacteriaceae (Family)										

50.15	*Ref.*	1.12 (0.91, 1.39)	0.95 (0.76, 1.19)	0.93 (0.74, 1.17)	0.74 (0.57, 0.97)	5.5E-04	0.021	0.93 (0.87, 1.0)	0.046	0.25
Firmicutes; Bacilli; Bacillales; Gemellaceae; Gemella (Genus)										
339.1	*Ref.*	0.78 (0.64, 0.95)	0.76 (0.62, 0.94)	0.7 (0.56, 0.87)	0.83 (0.65, 1.07)	9.4E-04	0.034	1.09 (1.02, 1.17)	0.0075	0.4
Actinobacteria; Coriobacteriia; Coriobacteriales; Coriobacteriaceae; Atopobium (Genus)										
47.04	*Ref.*	1.18 (0.96, 1.46)	0.97 (0.78, 1.21)	0.96 (0.76, 1.2)	0.82 (0.63, 1.06)	4.4E-04	0.031	0.93 (0.87, 1.0)	0.044	0.4
Firmicutes; Bacilli; Bacillales; Gemellaceae; Gemella; haemolysans (Species)										
237.49	*Ref.*	0.71 (0.56, 0.92)	0.73 (0.56, 0.95)	0.72 (0.55, 0.95)	0.74 (0.54, 1.01)	3.00E-04	0.039	1.12 (1.03, 1.22)	0.006	0.52
Actinobacteria; Actinobacteria; Actinomycetales; Actinomycetaceae; Actinomyces; viscosus (Species)										
1.34	*Ref.*	1.08 (0.72, 1.62)	0.92 (0.6, 1.42)	0.93 (0.59, 1.46)	1.09 (0.65, 1.83)	2.1E-04	0.039	0.79 (0.69, 0.90)	5.2E-04	0.14
Firmicutes; Negativicutes; Selenomonadales; Veillonellaceae; Selenomonas; noxia (OTU)										
0.9	*Ref.*	0.92 (0.55, 1.55)	1.54 (0.9, 2.64)	1.43 (0.82, 2.51)	1.16 (0.6, 2.23)	7.50E-05	0.023	0.72 (0.61, 0.85)	1.3E-04	0.04
Firmicutes; Bacilli; Bacillales; Gemellaceae; Gemella; haemolysans (OTU)										
237.95	*Ref.*	0.71 (0.55, 0.91)	0.73 (0.56, 0.95)	0.73 (0.55, 0.96)	0.74 (0.54, 1.01)	2.9E-04	0.036	1.12 (1.03, 1.22)	0.006	0.38
Actinobacteria; Actinobacteria; Actinomycetales; Actinomycetaceae; Actinomyces; viscosus (OTU)										
1.33	*Ref.*	1.08 (0.72, 1.63)	0.93 (0.61, 1.44)	0.95 (0.6, 1.48)	1.12 (0.67, 1.88)	3.5E-04	0.036	0.8 (0.697, 0.91)	8.1E-04	0.11

[a]Taxa included in the table were associated with daily carbohydrate or GI at q-trend <0.05 for categorical variables or q-value < 0.05 for continuous variables and were significant (q<0.05) in the full cohort analysis (n=834) at or below the family level

[b]DESeq2 models adjusted for age, sex, study (PLCOa, PLCOb, CPS-IIa, CPS-IIb,), current smoking, BMI (kg/m2), energy intake (kcal/day), and alcohol intake (grams/day).

[c]Trend tests across quintiles were calculated by entering the categorical variables into the models as continuous terms

[d]*P* not calculated due to heavy outlier influence on model (maximum Cook's distance > 10).

Supplementary Table S5(e). Association of daily carbohydrate and Glycemic Index (GI) as categorical (quintiles) or continuous variables with abundance of oral microbial taxa when restricting to PLCO and CPS-II cohort controls (n=543)[a]

PLCO and CPS-II Controls

		Categorical						Continuous		
**Mean normalized count**		**Fold change[b] (95% CI)**						**Fold change[b] (95% CI)**		
**Carbohydrates**	**Q1**	**Q2**	**Q3**	**Q4**	**Q5**	***P*-trend[c]**	**q-trend[c]**	**Per day**	***P***	**q-value**
Firmicutes (Phylum)										
8332.07	*Ref.*	0.79 (0.66, 0.94)	0.81 (0.68, 0.96)	0.79 (0.66, 0.94)	0.91 (0.76, 1.08)	0.003	0.021	0.99 (0.92, 1.07)	0.76	0.94
Actinobacteria; Coriobacteriia; Coriobacteriales (Order)										
51.08	*Ref.*	1.19 (0.89, 1.58)	0.96 (0.72, 1.26)	1.02 (0.77, 1.34)	0.87 (0.66, 1.15)	0.0071	0.13	0.83 (0.73, 0.93)	0.0021	0.04
Actinobacteria; Coriobacteriia; Coriobacteriales; Coriobacteriaceae (Family)										
48.74	*Ref.*	1.25 (0.93, 1.67)	1.02 (0.77, 1.35)	1.04 (0.78, 1.38)	0.94 (0.71, 1.26)	0.0023	0.074	0.81 (0.72, 0.92)	9.5E-04	0.031
Firmicutes; Bacilli; Lactobacillales; Streptococcaceae; Streptococcus; sp._oral_taxon_056 (Species)										
58.57	*Ref.*	0.86 (0.66, 1.13)	0.85 (0.65, 1.11)	0.89 (0.69, 1.16)	0.98 (0.75, 1.29)	5.1E-04	0.057	1.24 (1.11, 1.39)	2.2E-04	0.046
Firmicutes; Negativicutes; Selenomonadales; Veillonellaceae; Dialister; invisus (Species)										
5.16	*Ref.*	0.8 (0.55, 1.17)	0.81 (0.56, 1.16)	0.85 (0.59, 1.22)	0.85 (0.58, 1.22)	0.0067	0.25	0.76 (0.64, 0.89)	6.4E-04	0.046
Firmicutes; Bacilli; Lactobacillales; Streptococcaceae; Streptococcus; cristatus (Species)										
199.77	*Ref.*	0.99 (0.81, 1.21)	0.97 (0.79, 1.18)	0.91 (0.74, 1.1)	1.07 (0.88, 1.31)	0.0063	0.25	1.16 (1.07, 1.26)	6.1E-04	0.046
Firmicutes; Bacilli; Lactobacillales; Streptococcaceae; Streptococcus; sinensis (Species)										
29.37	*Ref.*	1.12 (0.82, 1.52)	0.86 (0.64, 1.16)	1.12 (0.83, 1.51)	1.11 (0.82, 1.5)	6.10E-05	0.013	1.14 (1.00, 1.3)	0.042	0.33
Firmicutes; Bacilli; Lactobacillales; Streptococcaceae; Streptococcus; sp._oral_taxon_056 (OTU)										
57.83	*Ref.*	0.85 (0.65, 1.12)	0.86 (0.66, 1.12)	0.9 (0.69, 1.16)	0.99 (0.76, 1.29)	5.00E-04	0.063	1.24 (1.11, 1.39)	1.5E-04	0.02
Firmicutes; Negativicutes; Selenomonadales; Veillonellaceae; Dialister; invisus (OTU)										
5.19	*Ref.*	0.76 (0.52, 1.1)	0.78 (0.54, 1.13)	0.8 (0.55, 1.15)	0.83 (0.57, 1.2)	0.0058	0.25	0.76 (0.64, 0.89)	7.0E-04	0.045
Firmicutes; Bacilli; Lactobacillales; Streptococcaceae; Streptococcus; cristatus (OTU)										
198.68	*Ref.*	0.99 (0.81, 1.2)	0.98 (0.81, 1.19)	0.91 (0.75, 1.11)	1.08 (0.89, 1.32)	0.0084	0.3	1.16 (1.07, 1.26)	5.2E-04	0.044
Firmicutes; Bacilli; Lactobacillales; Streptococcaceae; Streptococcus; sinensis (OTU)										
29.21	*Ref.*	1.1 (0.81, 1.49)	0.86 (0.64, 1.16)	1.11 (0.83, 1.49)	1.11 (0.82, 1.5)	7.30E-05	0.019	1.14 (1.01, 1.3)	0.041	0.37

Glycemic Index	Q1	Q2	Q3	Q4	Q5	*P*-trend[c]	q-trend[c]	Per day	*P*	q-value
-[e]										
-	*Ref.*	-	-	-	-	-	-	-	-	-

[a]Taxa included in the table were associated with daily carbohydrate or GI at q-trend <0.05 for categorical variables or q-value < 0.05 for continuous variables and were significant (q<0.05) in the full cohort analysis (n=834) at or below the family level

[b]DESeq2 models adjusted for age, sex, study (PLCOa, PLCOb, CPS-IIa, CPS-IIb,), current smoking, BMI (kg/m2), energy intake (kcal/day), and alcohol intake (grams/day).

[c]Trend tests across quintiles were calculated by entering the categorical variables into the models as continuous terms

[d]*P* not calculated due to heavy outlier influence on model (maximum Cook's distance > 10).

[e]No taxa met the q<0.05 threshold for significance

Supplementary Table S5(f). Association of daily carbohydrates as percent of calories as a categorical (quintiles) and continuous variable with abundance of oral microbial taxa (n=834)

	Mean normalized count	Categorical							Continuous		
		Fold change[b] (95% CI)							Fold change[b] (95% CI)		
**Carbohydrates as percent of calories**		**Q1**	**Q2**	**Q3**	**Q4**	**Q5**	***P*-trend[c]**	**q-trend[c]**	**Per day**	***P***	**q-value**
Fusobacteria; Fusobacteriia; Fusobacteriales; *Leptotrichiaceae; Leptotrichia* (Genus)											
	121.47	*Ref.*	0.98 (0.8, 1.21)	1.18 (0.95, 1.45)	1.12 (0.9, 1.38)	1.16 (0.94, 1.44)	3.70E-05	0.0026	1.02 (1.01, 1.02)	4.7E-05	0.0034
Fusobacteria; Fusobacteriia; Fusobacteriales; *Leptotrichiaceae; Leptotrichia; hongkongensis* (Species)											
	12.27	*Ref.*	1.03 (0.73, 1.47)	1.1 (0.77, 1.57)	1.27 (0.89, 1.83)	1.11 (0.78, 1.6)	0.0022	0.11	1.02 (1.01, 1.04)	0.00028	0.024
Firmicutes; Bacilli; Lactobacillales; *Streptococcaceae; Streptococcus; sp._oral_taxon_056* (Species)											
	59.06	*Ref.*	1.12 (0.9, 1.4)	1.27 (1.01, 1.59)	1.35 (1.07, 1.7)	1.5 (1.19, 1.88)	0.00087	0.088	1.02 (1.01, 1.02)	0.00023	0.024
Firmicutes; Bacilli; Lactobacillales; *Streptococcaceae; Streptococcus; cristatus* (OTU)											
	198.09	*Ref.*	1 (0.85, 1.17)	1.07 (0.91, 1.26)	1.24 (1.05, 1.46)	1.25 (1.06, 1.48)	0.00011	0.032	1.01 (1.01, 1.02)	0.00027	0.021
Actinobacteria; Actinobacteria; Actinomycetales; *Actinomycetaceae*; *Actinomyces*; *sp. oral taxon 180* (OTU)											
	22.4	*Ref.*	1.2 (0.82, 1.77)	1.23 (0.83, 1.83)	0.88 (0.59, 1.31)	0.88 (0.59, 1.31)	0.0018	0.089	0.97 (0.96, 0.98)	4.5E-05	0.014

[a]Taxa included in the table were associated with daily carbohydrate or GI at q-trend <0.05 for categorical variables or q-value < 0.05 for continuous variables and were significant (q<0.05) in the full cohort analysis (n=834) at or below the family level

[b]DESeq2 models adjusted for age, sex, study (PLCOa, PLCOb, CPS-IIa, CPS-IIb,), current smoking, BMI (kg/m2), and alcohol intake (grams/day).

[c]Trend tests across quintiles were calculated by entering the categorical variables into the models as continuous terms

[d]*P* not calculated due to heavy outlier influence on model (maximum Cook's distance > 10).

[e]No taxa met the q<0.05 threshold for significance

Supplementary Table S5(g). Association of daily Glycemic Load (GL), Sucrose, and Fiber intake as categorical (quintiles) and continuous variables with abundance of oral microbial taxa (n=834)[a]

Mean normalized count	Categorical							Continuous		
	Fold change[b] (95% CI)							Fold change[b] (95% CI)		
**Glycemic Load**	**Q1**	**Q2**	**Q3**	**Q4**	**Q5**	***P*-trend[c]**	**q-trend[c]**	**Per day**	***P***	**q-value**
Fusobacteria; Fusobacteriia; Fusobacteriales; Leptotrichiaceae (Family)										
122.38	*Ref.*	1.13 (0.92, 1.39)	1.21 (0.97, 1.5)	1.14 (0.91, 1.44)	1.13 (0.92, 1.39)	0.013	0.23	1 (1.002, 1.01)	7.7E-04	0.03
Fusobacteria; Fusobacteriia; Fusobacteriales; Leptotrichiaceae; Leptotrichia (Genus)										
22.36	*Ref.*	0.92 (0.62, 1.36)	0.87 (0.58, 1.31)	0.54 (0.35, 0.83)	0.92 (0.62, 1.36)	9.3E-06	0.003	0.99 (0.9987, 0.995)	3.4E-06	9.9E-04
Actinobacteria; Actinobacteria; Actinomycetales; Actinomycetaceae; Actinomyces; sp._oral_taxon_180 (OTU)										
121.33	*Ref.*	1.11 (0.9, 1.37)	1.2 (0.97, 1.49)	1.17 (0.93, 1.47)	1.11 (0.9, 1.37)	0.006	0.37	1 (1.002, 1.006)	2.5E-04	0.02
**Sucrose**	**Q1**	**Q2**	**Q3**	**Q4**	**Q5**	***P*-trend[c]**	**q-trend[c]**	**Per day**	***P***	**q-value**
-[e]										
-	*Ref.*	-	-	-	-	-	-	-	-	-
**Fiber**	**Q1**	**Q2**	**Q3**	**Q4**	**Q5**	***P*-trend[c]**	**q-trend[c]**	**Per day**	***P***	**q-value**
Firmicutes; Bacilli; Bacillales; Gemellaceae (Family)										
330.04	*Ref.*	1.22 (0.99, 1.5)	1.11 (0.91, 1.37)	1.3 (1.06, 1.6)	1.14 (0.92, 1.4)	0.087	0.3	0.98 (0.968, 0.995)	0.0078	0.037
Actinobacteria; Actinobacteria; Actinomycetales; Actinomycetaceae; Actinomyces; sp._oral_taxon_169 (Species)										
39.04	*Ref.*	0.85 (0.6, 1.21)	1.04 (0.73, 1.47)	1 (0.71, 1.42)	1.3 (0.92, 1.85)	0.066	0.39	1.04 (1.02, 1.07)	0.00034	0.017
Actinobacteria; Actinobacteria; Actinomycetales; Actinomycetaceae; Actinomyces; sp._oral_taxon_175 (Species)										
5.77	*Ref.*	0.63 (0.42, 0.95)	0.72 (0.48, 1.07)	0.67 (0.45, 0.99)	0.94 (0.63, 1.4)	0.0097	0.18	1.07 (1.04, 1.10)	2.1E-06	0.00034
Firmicutes; Negativicutes; Selenomonadales; Veillonellaceae; Dialister; pneumosintes (Species)										
1.78	*Ref.*	0.72 (0.44, 1.2)	0.82 (0.5, 1.35)	0.53 (0.32, 0.89)	0.87 (0.52, 1.43)	0.059	0.38	0.95 (0.91, 0.98)	0.0017	0.047
Actinobacteria; Actinobacteria; Actinomycetales; Actinomycetaceae; Actinomyces; sp._oral_taxon_169 (OTU)										
38.85	*Ref.*	0.83 (0.59, 1.18)	1.03 (0.73, 1.45)	0.99 (0.7, 1.4)	1.27 (0.9, 1.8)	0.079	0.5	1.04 (1.02, 1.07)	0.00053	0.027
Actinobacteria; Actinobacteria; Actinomycetales; Actinomycetaceae; Actinomyces; sp._oral_taxon_175 (OTU)										
2.98	*Ref.*	0.86 (0.55, 1.34)	1.19 (0.78, 1.84)	0.98 (0.64, 1.52)	1.45 (0.94, 2.23)	0.14	0.62	1.05 (1.02, 1.08)	0.0011	0.048

[a]Taxa included in the table were associated with daily carbohydrate or GI at q-trend <0.05 for categorical variables or q-value < 0.05 for continuous variables and were significant (q<0.05) in the full cohort analysis (n=834) at or below the family level
[b]DESeq2 models adjusted for age, sex, study (PLCOa, PLCOb, CPS-IIa, CPS-IIb,), current smoking, BMI (kg/m2), energy intake (kcal/day), alcohol intake (grams/day), carbohydrate intake (g/day), and GI.

[c]Trend tests across quintiles were calculated by entering the categorical variables into the models as continuous terms

[d]*P* not calculated due to heavy outlier influence on model (maximum Cook's distance > 10).

[e]No taxa met the q<0.05 threshold for significance
